# Dual‐Key Genetic Circuit Enables Stable and Self‐Regulated Engineered Bacteria for the Treatment of Ulcerative Colitis

**DOI:** 10.1002/advs.202520434

**Published:** 2026-02-13

**Authors:** Shuaijie Ding, Biao Yang, Xinyu Li, Yi Wang, Chunyu Huang, Wenfei Dong, Wei Xie

**Affiliations:** ^1^ Institute of Health and Medicine (IHM) Hefei Comprehensive National Science Center Hefei Anhui China; ^2^ Department of Oncology, Tongji Hospital, Tongji Medical College Huazhong University of Science and Technology Wuhan Hubei China; ^3^ Suzhou Institute of Biomedical Engineering and Technology Chinese Academy of Science (CAS) Suzhou China

**Keywords:** engineered bacteria, genetic circuit, quorum sensing, ulcerative colitis

## Abstract

Engineered bacteria offer a promising therapeutic platform but often display plasmid instability and antibiotic dependence. A synthetic dual‐key genetic circuit is established in *Escherichia coli* Nissle 1917 (EcN) by deleting both *asd* and *thyA* genes, generating an auxotrophic chassis that requires dual‐plasmid complementation for survival. The “lysis module” restores *asd* function and incorporates a quorum‐sensing system for self‐regulated lysis and controlled protein release (“One Key”). By contrast, the “expression module” complements *thyA* and co‐delivers an interleukin‐2 (IL‐2) mutant and the membrane protein Amuc_1100 to modulate immune balance and repair the intestinal barrier (“Dual Keys”). This dual‐key design enabled antibiotic‐free plasmid stability, precise population control, and sustained therapeutic protein secretion. Oral administration of the engineered strain significantly alleviated colitis in mice by enhancing regulatory T‐cell expansion, restoring epithelial integrity, and reshaping the gut microbiota. This modular system provides a safe, stable, and programmable strategy for live bacterial therapy against immune and mucosal diseases.

## Graphical abstract

A self‐regulated *Escherichia coli* Nissle 1917 strain equipped with a dual‐key genetic circuit achieved antibiotic‐free stability and programmable lysis for therapeutic protein delivery. By co‐expressing an IL‐2 mutant and Amuc_1100, the engineered bacteria restored immune balance and epithelial integrity, providing a safe and precise approach for treating ulcerative colitis.

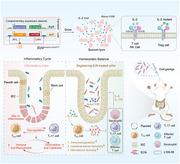

## 1 Introduction

Ulcerative colitis (UC) is a chronic and recurrent gastrointestinal disease, and conventional treatments have largely failed to resolve its core problems, including intestinal immune imbalance, barrier dysfunction, and gut microbiota dysbiosis [1]. Moreover, long‐term medication often causes severe adverse effects such as increased susceptibility to infections [2]. *EcN*, a generally recognized safe probiotic, has attracted significant attention owing to its unique biological properties. It can serve as a chassis for engineering, enabling the delivery of therapeutic genes or the synthesis of functional molecules [3, 4, 5, 6], thus offering potential applications in various diseases [7]. However, under metabolic stress in vivo, plasmids in engineered bacteria are prone to loss, which diminishes their therapeutic effectiveness. Current strategies for plasmid stabilization include the use of antibiotics or single‐gene‐defective plasmid‐based balanced lethal systems. Several classes of antibiotics, such as ampicillin, metronidazole, neomycin, and vancomycin, can impair the intestinal mucosal barrier, disrupt the gut microbiota, and hinder UC treatment [8]. This facilitates the acquisition of antibiotic resistance by pathogenic microorganisms and may compromise the efficacy of future antibiotic treatments [5, 9]. Additionally, single‐gene defective systems result in reduced plasmid retention rates, either due to nutritional compensation or horizontal gene transfer, limiting their long‐term effectiveness [10]. Dual‐gene defective systems, while more reliable, rely heavily on environmental nutritional compensation. This can impair bacterial growth [11, 12], and the lack of effective microbial density control leads to uncontrolled bacterial proliferation [13]. This may result in adverse outcomes such as disruption of the intestinal microbial balance, excessive immune responses, accumulation of metabolic toxicity, and compromised biosafety and therapeutic precision [14].

**FIGURE 1:**
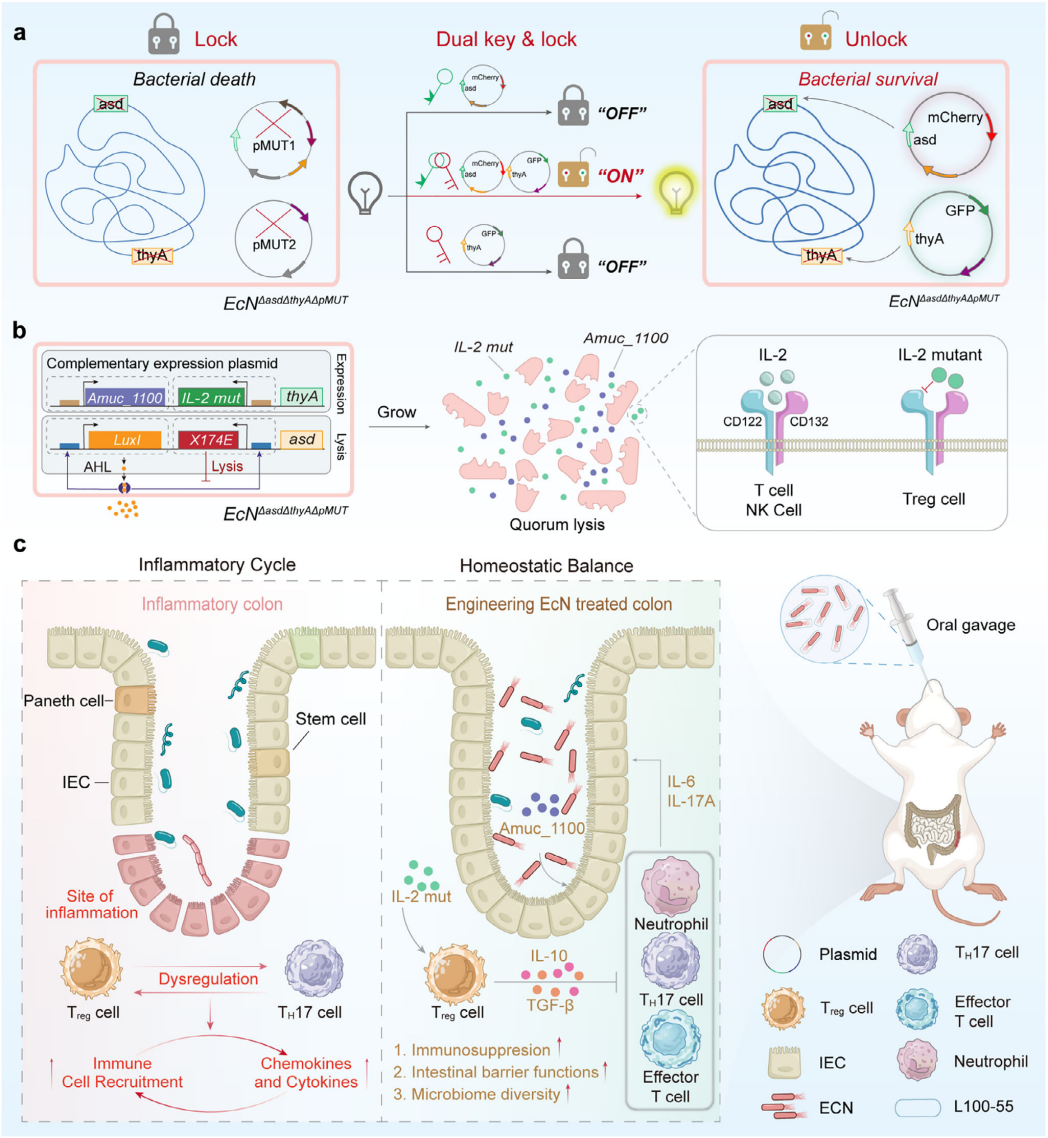
“Dual‐key” engineered bacteria for the treatment of UC. (a) Diagram of the dual‐plasmid knockout system and endogenous plasmid knockout, with GFP‐*thyA* and mCherry‐*asd* used for compensation. When both plasmids are present, the plasmids survive and express GFP and mCherry. (b) Dual compensatory plasmid integration expression module and lysis module compensate for *EcN* host defects. SLC‐driven strains undergo lysis, releasing IL‐2 mutants and Amuc_1100, with IL‐2 mutants enhancing Treg activation. (c) L100‐55 encapsulated probiotics resist gastric acid and colonize the mouse intestine, releasing IL‐2 mutants and Amuc_1100 in the intestine, balancing Treg and Th17 immune cells, promoting barrier repair, and used for the treatment of UC.

Previous studies have shown that *asd* encodes a key enzyme in diaminopimelic acid (DAP) synthesis, which is a crucial component of the Gram‐negative bacterial cell wall. The loss of DAP leads to rapid bacterial lysis [15, 16]. Similarly, *thyA* encodes thymidylate synthase and its deficiency impairs thymidine synthesis, thereby impairing DNA replication, halting cell division, and causing bacterial death due to nucleic acid metabolic disorders [10, 17]. Using λ‐Red recombination [18], we created a double‐auxotrophic *EcN* strain with knockout mutations in both *asd* and *thyA*, rendering it unable to survive independently [12, 19]. By introducing dual plasmids that carry complementary genes, this “dual‐key” plasmid‐compensated biosafety system ensures bacterial survival and allows for efficient antibiotic‐independent expression. The engineered bacteria can survive and express functional proteins only when both plasmids are present (the “ON” state, with expression of red and green fluorescent signals). If either plasmid is lost, the nutrient‐synthesis pathways are disrupted, causing bacterial collapse or failure to divide, leading to cell death (the “OFF” state, with no fluorescent signal) (Figure 1a). The compensatory plasmid that expresses the *asd* gene integrates into a synchronized lysis circuit (SLC). SLC uses the LuxI/R quorum‐sensing system to regulate the phage lysis gene *ϕX174E*, enabling engineered bacteria to initiate self‐lysis once their intestinal colonization reaches a critical density [20, 21, 22]. This approach facilitates the pulsatile release of functional proteins as residual bacteria regrow and undergo repeated growth–lysis cycles, thereby maintaining stable local protein concentrations. It minimizes fluctuations and systemic toxicity associated with conventional systemic administration while preventing uncontrolled bacterial overgrowth [23, 24]. The *thyA*‐based compensatory plasmid co‐expresses the target protein, ensuring effective expression and delivery of the proteins for functional repair (Figure 1b).

Most current clinical treatment approaches to UC focus primarily on alleviating symptoms by suppressing intestinal inflammation [25, 26, 27]. However, these methods often fail to address the root causes of the disease, such as the vicious cycle of immune imbalance and barrier dysfunction, and cannot resolve both inflammatory activation from pathogen invasion and ongoing barrier impairment [28, 29]. Restoring the immune balance requires an increase in regulatory T cells (Tregs) and a reduction in T helper 17 (Th17) cells, which induce immune suppression [30, 31]. Interleukin (IL)‐2 is a key factor in the survival and function of Tregs, because these cells express high‐affinity IL‐2 receptors (CD25, CD122, and CD132). By binding to these receptors, IL‐2 activates downstream signaling pathways that promote Treg survival and proliferation [32]. However, high concentrations of IL‐2 can also non‐selectively activate effector T and Natural Killer (NK) cells, leading to systemic immune activation. We previously used IL‐2 mutants that specifically activate Treg receptors and avoid effector cell stimulation to mitigate these side effects [33, 34]. For barrier repair, the membrane protein Amuc_1100 from *Akkermansia* enhances the expression of tight junction proteins in intestinal epithelial cells [35, 36]. However, most current treatments lack a synergistic approach that simultaneously targets immune regulation and barrier repair, leaving the immune imbalance and barrier damage unresolved [37, 38].

In this study, we engineered a compensatory plasmid encoding *thyA* to co‐express an IL‐2 mutant and the Amuc_1100 protein. The IL‐2 mutant (N103R/V106D) selectively activates the Treg‐specific CD25 receptor, suppressing effector T and NK cell activity to restore the immune balance. Amuc_1100 promoted the expression of epithelial tight junction proteins, strengthened the intestinal barrier, and enabled dual intervention in immune regulation and barrier repair (Figure 1b,c). The CRISPR/Cas9 technology was used to eliminate the cryptic plasmids pMUT1 and pMUT2 from *EcN*, thereby increasing the expression of exogenous proteins, to enhance the expression of exogenous genes and improve plasmid compatibility [39]. Additionally, the L100‐55 enteric coating protects engineered bacteria from gastric acid, ensuring the targeted delivery of active components to colonic lesion sites, thereby enhancing stability and targeting within the intestinal environment and preventing acid‐induced damage [40]. This modular design system is adaptable to different chassis strains and disease scenarios and offers promising potential for expanding engineered bacterial therapies to treat a variety of diseases.

## 2 Results

### 2.1 Effect of Antibiotics on the Intestinal Flora

**FIGURE 2:**
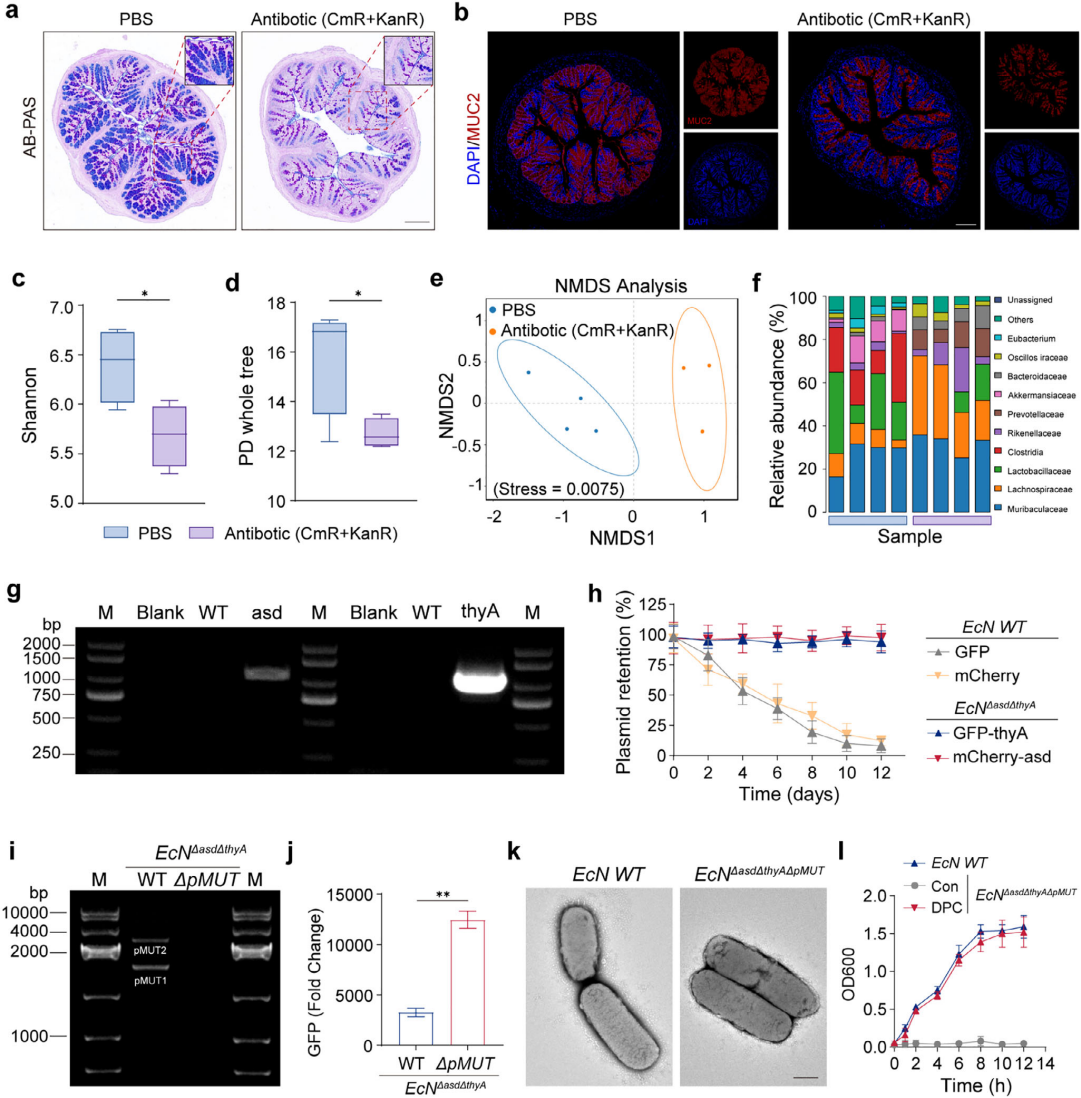
Antibiotic effects and dual compensation system construction. (a,b) Representative images of AB–PAS staining (a) and immunofluorescence staining (b) of MUC2 (red) and DAPI (4',6‐diamidino‐2‐phenylindole, blue) in the proximal colon of mice following oral treatment with chloramphenicol and kanamycin (CmR+KanR). Scale bar, 500 µm. (c,d) 16S rRNA sequencing of mouse feces after CmR+KanR treatment for strain diversity analysis: Shannon's index (c), and PD (d). (e,f) NMDS plot based on gut microbial community sequencing data (e), with colored ellipses representing the distribution of each of the two sample groups. Relative abundance of microbial taxa (f). (g) Agarose gel electrophoresis of *EcN* strains with *asd* and *thyA* gene knockouts generated via λ‐Red recombineering, showing distinct banding patterns. (h) Wild‐type *EcN* transformed with normal plasmids and host‐defective strains containing dual‐compensated fluorescent plasmids (GFP‐*thyA* and mCherry‐*asd*). (i,j) Cas9 knockout of endogenous plasmids pMUT1 and pMUT2: agarose gel electrophoresis results (i) and analysis of GFP expression (j). (k,l) Transfection of dual‐compensated fluorescent plasmids into a double auxotrophic strain: electron microscopy images of strain morphology (k) and OD measurements of growth curves (l). Scale bar, 200 nm. Data are presented as mean ± SEM (*n* = 4 biologically independent samples for (c–f); *n* = 3 biologically independent samples for (h, j, and l)). Statistical significance was calculated relative to the control group (c, d, k). *P* values were determined using Student's two‐sided *t*‐test (*p < 0.05, **p < 0.01, ***p < 0.001, and ****p < 0.0001).

*EcN*, an engineered bacterial vector used for gene delivery, has shown significant therapeutic potential [41, 42]. However, exogenous plasmid insertion can lead to plasmid loss under metabolic stress, thereby reducing therapeutic efficacy. Although antibiotics are commonly used to maintain plasmid stability, they can disrupt the gut microbiota and intestinal barrier [43]. In this study, a mixture of chloramphenicol and kanamycin (CmR+KanR) was administered to mice and colonic mucosal proteins were analyzed. AB–PAS and immunofluorescence staining of MUC2 revealed reduced colonic mucosal protein levels, whereas hematoxylin and eosin (H&E) staining revealed no significant changes. A reduction in colonic barrier protein levels was also observed (Figure 2a,b; Figure S1). Fecal samples were collected for 16S rRNA sequencing and microbial diversity and community structure determination to assess the impact of antibiotics on the gut microbiota (Figure 2c–f). α‐Diversity analysis showed that the Shannon index and phylogenetic diversity (PD whole tree) were significantly lower in the antibiotic‐treated group than in the phosphate buffer saline (PBS) control group, indicating reduced species richness, evenness, and evolutionary diversity. Non‐metric multidimensional scaling (NMDS) analysis confirmed that the two groups formed distinct clusters, demonstrating that the antibiotics reshaped the microbial community structure. Therefore, antibiotics are unsuitable for maintaining plasmid stability in vivo during UC treatment.

### 2.2 Dual‐Key System Construction and Characterization

λ‐Red recombination technology was used to delete the *asd* and *thyA* genes from the *EcN* genome, resulting in the construction of the *EcN^ΔasdΔthyA^* strain (Figure 2g). We engineered compatible plasmids expressing GFP and mCherry to compensate for the loss of *asd* and *thyA* expression. The genes for ampicillin and chloramphenicol resistance in these plasmids were deleted to ensure safety. Under long‐term antibiotic‐free conditions, the plasmids showed high retention rates, and the fluorescence intensity remained strong (Figure 2h; Figure S2). The wild‐type *EcN* strain contains endogenous plasmids pMUT1 and pMUT2, which hinder the introduction and stable expression of exogenous plasmids [39]. Cas9‐mediated knockout of these plasmids was performed to enable exogenous gene expression to overcome this limitation (Figure 2i). GFP fluorescence analysis demonstrated that the knockout of endogenous plasmids significantly enhanced GFP expression (Figure 2j). Bacterial growth curves and morphological characteristics were analyzed to assess the impact of genetic modifications on bacterial growth and morphology. The results indicated that the dual‐plasmid system did not affect the bacterial growth or morphology (Figure 2k,l). These in vitro experimental results confirmed the feasibility of efficiently expressing exogenous plasmids using the dual‐plasmid deletion system, providing critical preliminary evidence for subsequent in vivo studies that will assess the retention capacity of the dual‐plasmid system and its corresponding therapeutic efficacy in the in vivo physiological milieu.

### 2.3 Configuration of SLC and Functional Analysis of Proteins

**FIGURE 3:**
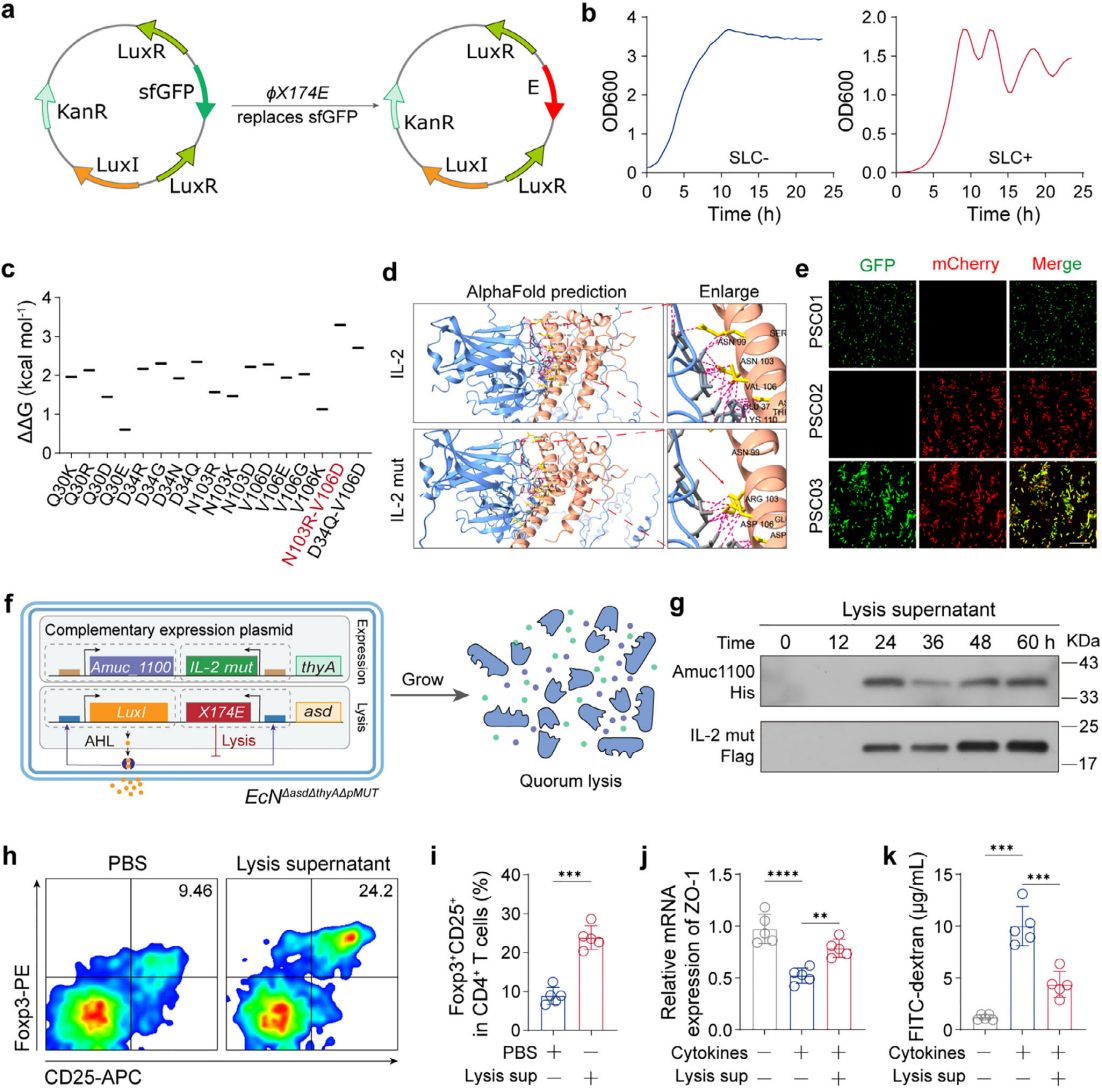
Configuration of SLC and functional analysis of proteins. (a) Schematic diagram of SLC plasmid construction, *ϕX174E* replacing sfGFP in pTD103. (b) Bacterial growth dynamics over time of SLC+ and SLC− *EcN* in liquid culture. (c) MutaBind2 simulates the binding energy between different IL‐2 mutants and CD122. Red markings indicate the highest reduction in binding energy. (d) AlphaFold2 simulates the interaction between IL‐2 and CD122. Enlarge indicates local magnification, and the red arrow indicates a reduction in interaction sites. (e) Fluorescence expression images were detected using a confocal microscope, including J23119‐GFP (PSC01), tac‐mCherry (PSC02), and J23119‐GFP‐tac‐mCherry (PSC03) driven fluorescence expression, respectively. (f) Schematic diagram of a dual‐plasmid compensation system containing expression plasmids and lysis plasmids in engineered bacteria with host gene defects. (g) Supernatants from engineered bacteria with dual‐compensated plasmid systems were collected at regular intervals in vitro, and Flag and His‐tagged proteins were detected using Western blot. (h,i) Tregs (CD25^+^Foxp3^+^) were detected using flow cytometry (h) and statistically analyzed (i) after spleen stimulation with lysis supernatant. (j,k) Caco‐2 damage cell model treated with lysate supernatant. ZO‐1 mRNA levels were detected using quantitative PCR (j), and Caco‐2 monolayer permeability was assessed using FITC‐dextran assay (k). Data are presented as mean values ± SEM (*n* = 5 biologically independent samples for (i–k)). Statistical significance was determined by comparing with the PBS group. *P* values were determined using Student's two‐sided *t*‐test (*p < 0.05, **p < 0.01, ***p < 0.001, and ****p < 0.0001).

SLC technology has been applied to efficiently deliver functional proteins while preventing uncontrolled proliferation of engineered bacteria [20, 21, 22, 23, 24, 44]. This technology, extensively used in tumor immunotherapy, achieves periodic bacterial lysis by replacing the *sfGFP* gene with the phage *ϕX174E* gene on the pTD103 plasmid (Figure 3a; Figure S3). The lysis cycle was monitored by measuring bacterial concentration via OD600. Figure 3b shows the periodic growth of engineered bacteria carrying the SLC plasmid with flocculent material in the culture medium (Figure S4). This material released functional proteins and prevented excessive bacterial proliferation in the gut microbiota.

For protein selection, IL‐2 and Amuc_1100 were selected to target immune balance and barrier repair. IL‐2 mutants (N103R and V106D) selectively activated Tregs by reducing CD122 affinity, whereas higher concentrations activated T and NK cells. MutaBind2 simulations revealed that the N103R and V106D mutants had significantly reduced IL‐2 binding affinity for CD122 (Figure 3c). The AlphaFold2 simulations further confirmed this reduction [45] (Figure 3d). Amuc_1100 promotes epithelial cell repair and immunosuppressive factor production. A dual‐expression plasmid containing the J23119‐GFP and tac‐mCherry promoters was constructed to co‐express both proteins. Confocal microscopy confirmed the independent expression of fluorescent proteins (Figure 3e), with Amuc_1100 and IL‐2 mutants replacing mCherry and GFP, respectively. Western blotting, ELISA, and immunofluorescence confirmed the protein expression (Figures S5 and S6).

The SLC plasmid with the DAP compensatory gene and the *thyA* plasmid were co‐transformed into *EcN^ΔasdΔthyAΔpMUT^*. Once the bacterial concentration reached a threshold, lysis occurred, releasing the proteins (Figure 3f). Growth curve analysis confirmed the periodic bacterial growth (Figure S7). Supernatant analysis revealed a persistent protein release (Figure 3g; Figure S8). Flow cytometry demonstrated that the IL‐2 mutants induced Treg activation without activating T or NK cells (Figure 3h,I; Figure S9). In the Caco‐2 cell model, the lysate supernatant enhanced ZO‐1 expression and reduced intestinal permeability

In the Caco‐2 cell model, the supernatant from engineered bacterial lysate enhanced ZO‐1 mRNA expression. Concurrently, Fluorescein Isothiocyanate (FITC)‐dextran assays demonstrated that the supernatant reduced intestinal permeability (Figure 3j,k; Figure S10). These results suggest that the engineered bacteria can specifically activate Tregs, promote barrier repair, and reduce intestinal permeability.

### 2.4 Engineered Bacteria Modification for Long‐Term Colonization in the Intestine

**FIGURE 4:**
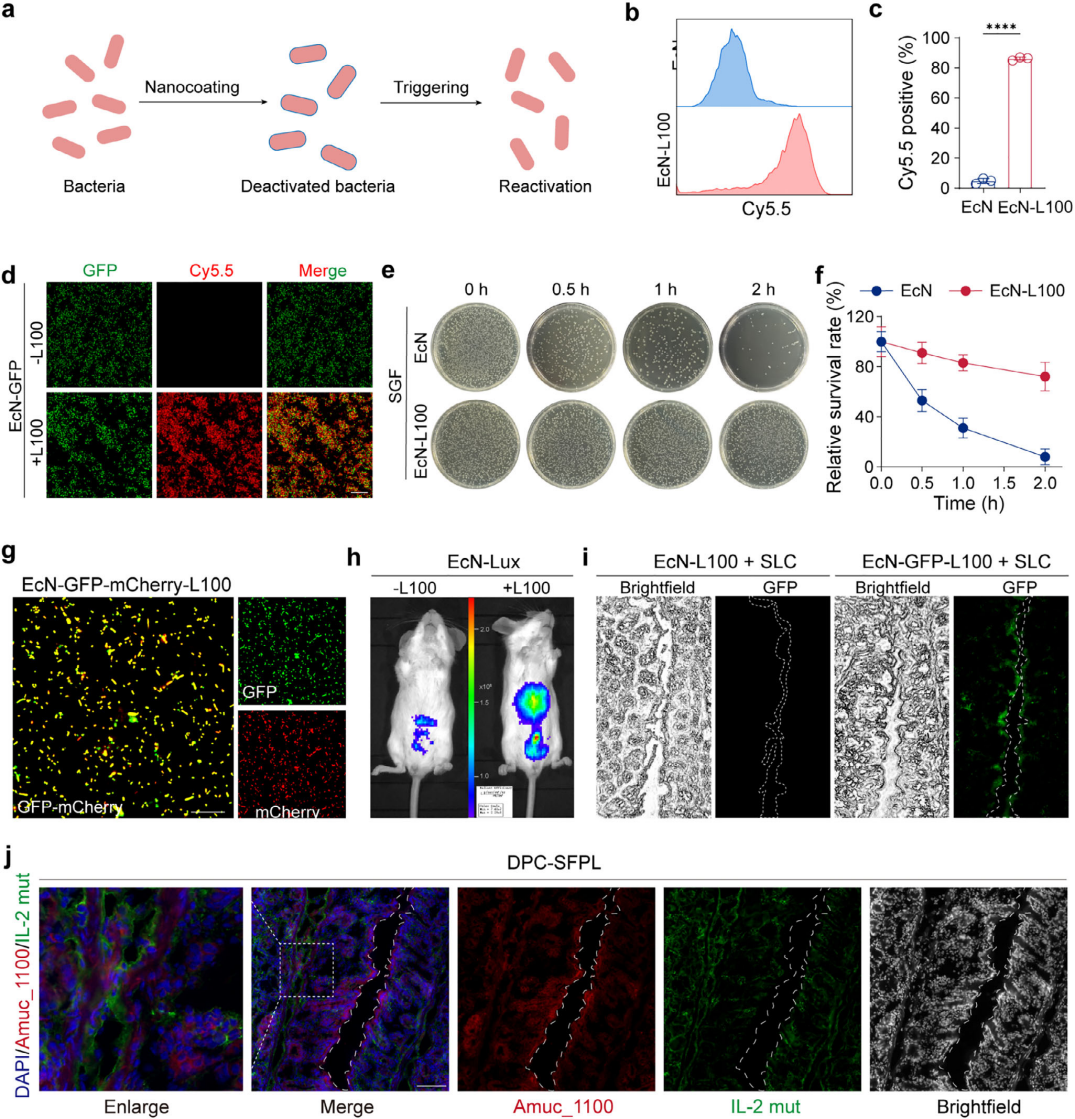
Characterization of Engineered Bacteria and Detection of Prolonged Colonization. (a) Schematic diagram of the modification and stimulation of bacterial expression. (b,c) Flow cytometry histogram (b) and quantitative statistics graph (c) of *EcN* and L100‐Cy5.5 encapsulated *EcN*. (d) Representative images of Cy5.5 fluorescence signals detected using L100‐Cy5.5 encapsulated *EcN*‐GFP or *EcN*‐GFP confocal microscopy. Scale bar, 50 µm. (e,f) Bacteria were collected at regular intervals and spread on resistant plates. Resistance plate images (e) and survival rates (f) were obtained for *EcN* and *EcN*‐L100 in simulated gastric acid. (g) Image of bacterial autofluorescence expression after confocal detection of L100 encapsulated with *EcN*‐GFP‐mCherry. Scale bar, 10 µm. (h) Bioluminescence images of mice after oral gavage of uncoated *EcN*‐Lux or *EcN*‐Lux‐L100 for 24 h. (i) Oral administration of SLC‐*EcN*‐GFP‐L100 and detection of GFP fluorescence in colon sections. (j) Representative immunofluorescence images of IL‐2 mut (green) and Amuc_1100 (red) in the proximal colon at 24 h after DPC‐SFPL gavage treatment. Cell nuclei were labeled with DAPI (blue). Scale bar, 50 µm. Data are presented as mean values ± SEM (*n* = 3 biologically independent samples for (c, f, and h–k)). Statistical significance was determined by comparing the results with those of the PBS group (c, k). *P* values were determined using Student's two‐sided *t*‐test (*p < 0.05, **p < 0.01, ***p < 0.001, and ****p < 0.0001).

Orally engineered bacteria are prone to damage from stomach acid. Bacteria were encapsulated in L100‐55, a methyl acrylate‐ethyl acrylate copolymer (referred to as L100), which enables pH‐triggered de‐encapsulation in the intestine to address this issue (Figure 4a). This strategy protects the bacteria from acid damage during gastric transit and restores their activity once they reach the intestine [40]. Under acidic conditions, *EcN*‐L100 maintains the structural integrity and biological activity of *EcN*, whereas the polymer matrix dissolves under neutral conditions. Cy5.5 was conjugated to L100 to confirm the success of L100 modification. Flow cytometry showed that L100‐Cy5.5 *EcN* had significantly higher fluorescence intensity (Figure 4b,c), and confocal microscopy revealed L100 encapsulation on the surface of *EcN*‐GFP strains (Figure 4d). Morphological changes were observed, but the fluorescence expression remained intact (Figures S11 and S12).

In vitro, *EcN*‐L100 was incubated with simulated gastric fluid (SGF). The results showed that *EcN*‐L100 maintained its intact morphology and had high survival rates, indicating that L100 protected the strain from gastric acid damage (Figure 4e,f). L100 encapsulation did not affect plasmid‐encoded protein expression in *EcN* cells (Figure 4g). The strains were incubated in SGF and bile salt solutions to assess the protein release in the intestine. ELISA detected higher protein levels in the bile salt solution, confirming that the engineered bacteria released functional proteins into the intestinal environment (Figure S13).

In vivo, mice that were orally administered *EcN*‐Lux or *EcN*‐Lux‐L100 showed significant bioluminescence in their intestines, indicating protection from gastric acid (Figure 4h; Figure S14). Fluorescent signals were observed in the colon to verify whether the protein was released into the colon after the SLC‐mediated lysis of the engineered bacteria (Figure 4i). Additionally, colonic tissues from mice treated with Dual Plasmid Compensation SLC Functional Protein‐L100 (DPC‐SFPL) in *EcN^ΔasdΔthyAΔpMUT^* were examined to confirm whether the IL‐2 mutant and Amuc_1100 can be expressed and released in the intestine. The immunofluorescence results showed that both the IL‐2 mutant and Amuc_1100 were effectively expressed in the intestine (Figure 4j). PCR amplification of the target gene fragment indicated that the strain colonized mouse intestines for an extended period, demonstrating high stability (Figures S15 and S16). These findings suggest that *EcN*‐L100 effectively protects *EcN* from gastric acid–induced degradation and facilitates targeted protein release in the intestine.

### 2.5 Biocompatibility of Engineered Bacteria

The biosafety of DPC‐SFPL was assessed before its application in the UC model. In this experiment, the engineered bacteria were administered orally on days 3, 5, and 7 (1 × 10^8^ CFU per mouse). Serum biochemical tests and complete blood counts were performed on day 10 (Figures S17 and S18) and revealed no significant abnormalities. Peripheral blood samples were collected at regular intervals and cultured on antibiotic‐resistant agar plates. No significant bacterial colonies were observed (Figure S19), indicating that the engineered bacteria did not enter the peripheral blood. Furthermore, compared with healthy mice, those treated with DPC‐SFPL showed no significant differences in the major organ tissue sections (Figure S20). These findings suggest that DPC‐SFPL does not induce adverse reactions and exhibits sustained efficacy.

### 2.6 Engineering Bacteria to Repair the Intestinal Barrier in the Dextran Sulfate Sodium Model

**FIGURE 5:**
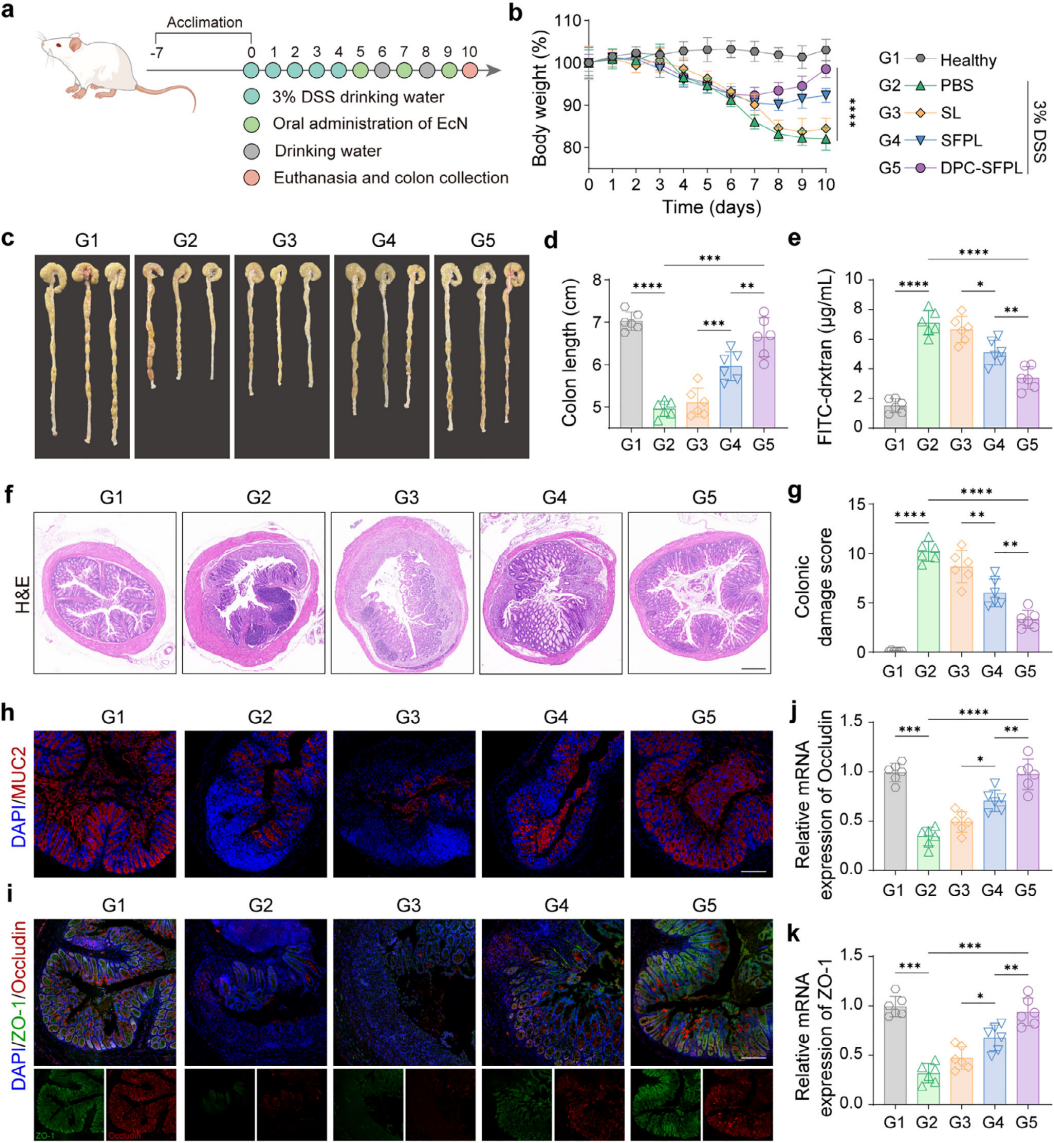
Role of engineered bacteria in barrier repair in the DSS model. (a) Experimental design of in vivo assessment using a DSS‐induced model. Mice were pretreated with PBS, SL, SFPL, and DPC‐SFPL. (b) Changes in mouse body weight following treatment. (c,d) Digital photographs of colon samples (c) and average colon lengths (d) from treated mice. (e) Intestinal barrier integrity was assessed using a FITC‐dextran assay after treatment with different groups. (f,g) Representative H&E‐stained colon tissue images (f) and colonic damage scores (g) from treated mice. Scale bar, 500 µm. (h) Immunofluorescence staining of MUC2 (red) in the proximal colon sections. Nuclei were stained with DAPI (blue). Scale bar, 100 µm. (i) Immunofluorescence images showing Occludin (red) and ZO‐1 (green) expression in the proximal colon. Nuclei were stained with DAPI (blue). Scale bar, 100 µm. (j,k) Relative expression levels of Occludin mRNA (j) and ZO‐1 mRNA (k) were detected using fluorescence qPCR in the proximal colon of mice from different treatment groups. Data are presented as mean values ± SEM (*n* = 6 biologically independent samples for (b, d, e, g, j, and k)). Statistical significance was determined using one‐way analysis of variance (ANOVA) with post hoc Tukey's correction for multiple comparisons (*p < 0.05, **p < 0.01, ***p < 0.001, and ****p < 0.0001).

In the Dextran Sulfate Sodium (DSS)‐induced UC model, the therapeutic efficacy and intestinal barrier repair capacity of the engineered bacterium, DPC‐SFPL, were evaluated. The UC model was established in BALB/c mice following a 7‐day adaptation period with free access to acidified water (pH 2.5) containing 3% DSS (Figure 5a). Three different strains were administered orally every 2 days, and the mouse body weight was continuously monitored. Compared with the healthy group, DSS‐treated mice exhibited various UC symptoms, including weight loss, elevated disease activity index (DAI), colon shortening, and tissue damage (Figure 5), confirming the successful establishment of the UC model. Mice with acute UC were subsequently administered PBS, SL (SLC‐L100 in *EcN*), SFPL (SLC Functional Protein‐L100 in *EcN*), or DPC‐SFPL (orally via gavage on days 5, 7, and 9, and 1 × 10^8^ CFU per mouse). Compared with the DSS model group, DPC‐SFPL treatment significantly reduced DSS‐induced UC symptoms, including weight loss and DAI, promoted colon length recovery, and reduced colon tissue damage (Figure 5b; Figure S21a). Colonic permeability assessed using FITC‐dextran perfusion revealed that serum concentrations in mice treated with DPC‐SFPL were significantly reduced compared with those in both the DSS model group and the SL group, approaching the levels observed in normal mice. This indicates that the intestinal barrier function was restored in DSS‐induced colitis (Figure 5e). Histopathological analysis of the colon tissue with H&E staining revealed well‐preserved finger‐like crypt structures and restored tissue integrity in the DPC‐SFPL group (Figure 5f,g). This suggests that DPC‐SFPL effectively promotes intestinal tissue repair in mice. MUC2, Occludin, and ZO‐1 maintain intestinal barrier integrity and functional homeostasis. Immunofluorescence analysis of the colon tissue revealed that MUC2 expression was restored in the SFPL and DPC‐SFPL groups compared with the DSS model and SL groups, whereas Occludin and ZO‐1 levels were elevated. The dual‐plasmid compensation strain DPC‐SFPL demonstrated superior barrier repair effects compared with those of the SFPL group, further validating the therapeutic advantages of the dual‐plasmid compensation system for intestinal treatment (Figure 5h,i). Additionally, the mRNA levels of Occludin and ZO‐1 (Figure 5j,k) were consistent with the immunofluorescence results. These results indicate that DPC‐SFPL treatment promotes the formation of tight junction complexes in the colon tissue, thereby enhancing barrier repair.

### 2.7 Immunologic Mechanisms of Engineered Bacteria in the Treatment of UC

**FIGURE 6:**
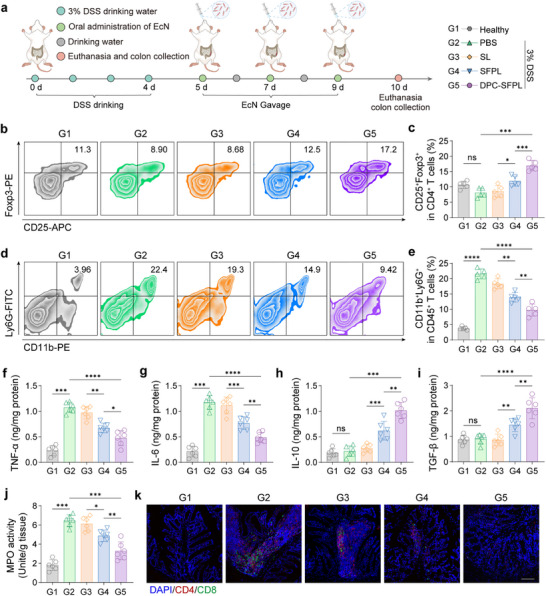
Immunologic mechanisms of engineered bacteria in the treatment of UC. (a) Schematic diagram of the experimental protocol for treating UC in mice with DSS. (b–e) Flow cytometry analysis of Tregs (b) (CD25^+^Foxp3^+^) and neutrophils (d) (CD11b^+^Ly6G^+^) in the colonic lamina propria of mice treated with different engineered bacteria. Statistical analysis of Tregs (c) and neutrophils (e) is shown. (f–i) Levels of TNF‐α (f), IL‐6 (g), IL‐10 (h), and TGF‐β (i) in the colon tissues measured using ELISA on day 10. (j) MPO activity in the colons of mice after different treatments. (k) Representative immunofluorescence images of CD4 (red) and CD8 (green) of the proximal colon of treated mice. Cell nuclei were stained with DAPI (blue). Scale bar, 100 µm. Data are presented as mean values ± SEM (*n* = 5 biologically independent samples for (c, e), *n* = 6 biologically independent samples for (f–j)). Statistical significance was determined using one‐way ANOVA with post hoc Tukey's correction for multiple comparisons across groups (*p < 0.05, **p < 0.01, ***p < 0.001, and ****p < 0.0001).

Previously, it was confirmed that colonic barrier repair was more effective following treatment with engineered bacteria, and its immunological mechanisms were explored (Figure 6a). IL‐2, as shown in previous studies, plays a key role in promoting and maintaining Tregs, which are specialized T cells involved in regulating immune responses [32]. The number of Tregs in the colon of mice after different treatments was measured. As shown in Figure 6b,c, after treatment with multiple sets of engineered bacteria, the SFPL and DPC‐SFPL groups exhibited increased numbers of Tregs in colonic lamina propria and reduced levels of splenic inflammation compared with those in the DSS model and SL groups. Furthermore, the DPC‐SFPL group demonstrated superior efficacy to that of the SFPL group, further indicating the advantages of the dual‐plasmid deletion system (Figures S22 and S23). In addition to Tregs, neutrophils exacerbate intestinal mucosal inflammation by releasing proinflammatory cytokines, and NET formation can damage the intestinal epithelial barrier and induce local immune imbalance [46]. Therefore, we analyzed neutrophils in the colonic lamina propria and found that following DPC‐SFPL treatment, the number of neutrophils (CD11b^+^ Ly6G^+^) was significantly reduced compared with that in the DSS model group (Figure 6d,e; Figure S24), indicating that the colon tissue was immunosuppressed post‐engineered bacterial treatment. The activation and recruitment of neutrophils are associated with IL‐17a secreted by Th17 cells. The expression levels of proinflammatory‐related genes were analyzed to further explore this. Compared with the DSS model group, transcription levels of IL‐17a, RORγt, and IL‐6 in colon tissue were significantly reduced after DPC‐SFPL treatment, whereas transcription of Foxp3 was increased (Figure S21b–e). Cytokine levels in the colon tissue were measured using ELISA. Following DPC‐SFPL treatment, levels of proinflammatory factors TNF‐α and IL‐6 were significantly reduced compared with those of the DSS model and SL groups (Figure 6f,g), whereas levels of immunosuppressive molecules TGF‐β and IL‐10 were significantly elevated in the same comparisons (Figure 6h,i). Myeloperoxidase (MPO) levels, which are elevated in the intestinal tissues of UC model animals, are correlated with the severity of intestinal inflammation. Compared with the DSS‐induced models, MPO levels were significantly reduced in the DPC‐SFPL treatment group, indicating reduced intestinal injury (Figure 6j). Additionally, colonic immunofluorescence revealed a significant reduction in CD8^+^ T–cell infiltration (Figure 6k). Similar therapeutic effects were observed when engineered bacteria were used during DSS administration (Figure S25). A 2,4,6‐trinitrobenzenesulfonic acid (TNBS)‐induced CD model was established to validate the therapeutic efficacy of this system in multiple IBD models. These results demonstrated that the DPC‐SFPL group exhibited favorable effects on colonic repair. (Figure S26). These results suggest that engineered bacteria can induce immune suppression in the colon and restore immune balance.

### 2.8 Engineering Bacteria to Regulate the Gut Microbiota

UC pathogenesis is linked to dysbiosis of the gut microbiota. A healthy microbiota suppresses inflammation by maintaining the immune balance, synthesizing metabolites, and protecting the intestinal barrier. In contrast, patients with UC have reduced microbial diversity, increased levels of proinflammatory pathogens, and fewer commensals. Dysbiosis compromises the intestinal barrier, allowing pathogen‐associated molecular patterns to enter the submucosa and trigger immune responses and chronic inflammation. We evaluated the efficacy of DPC‐SFPL in mice pretreated with antibiotics or drinking water, as well as in DSS‐induced acute UC models, to investigate the role of the gut microbiota. The results showed that DPC‐SFPL demonstrated good therapeutic efficacy in mice treated with drinking water; however, its therapeutic effect was significantly reduced in antibiotic‐treated mice. This indicates that antibiotics can alter the gut microbiota, thereby affecting the therapeutic efficacy of engineered bacteria (Figure S27). This highlights the role of the microbiota in acute UC and warrants further investigation into the regulation of microbial abundance by DPC‐SFPL.

**FIGURE 7:**
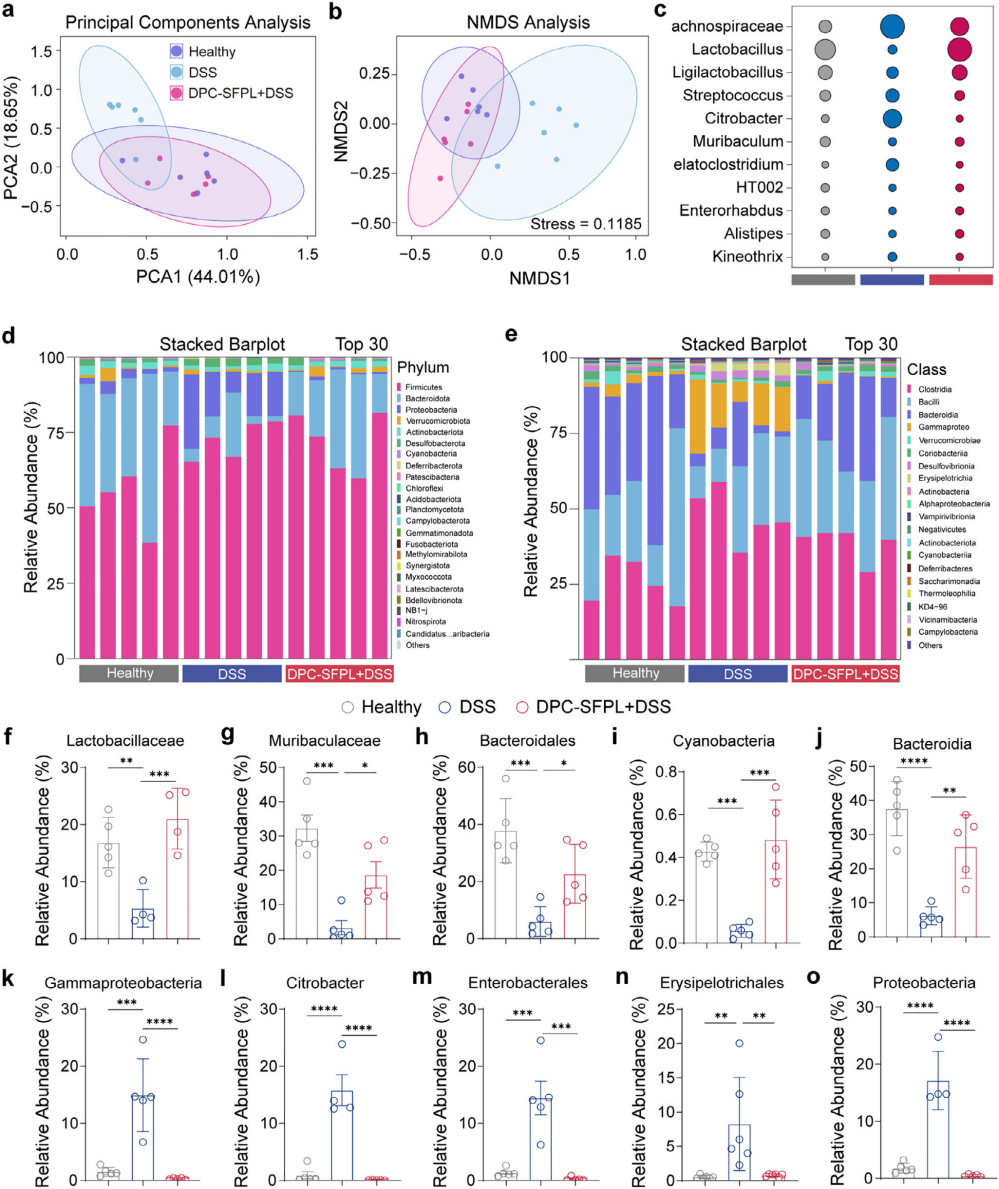
Engineered bacteria regulate the intestinal microbiota. (a) PCA showed the differences in sample distribution among the healthy (purple), DSS (blue), and DPC‐SFPL (pink) groups. (b) NMDS analysis verified the differences in community structure. (c) Bacterial genus abundance bubble chart, with color gradients characterizing the relative abundance of different bacterial genera. (d) Horizontal stacked bar chart of the door shows the composition of the flora in the top 30 abundances. (e) Horizontal stacked bar chart of the class reveals the differences in microbiota classification. (f–o) Histogram of the relative abundance of gut microbiota. Data are presented as mean values ± SEM (*n* = 5 biologically independent samples for (d–o)). Statistical significance was determined by comparing with the PBS group. *P* values were determined using Student's two‐sided *t*‐test (*p < 0.05, **p < 0.01, ***p < 0.001, and ****p < 0.0001).

DSS‐induced UC was established in BALB/c mice, followed by PBS or DPC‐SFPL (1 × 10^8^ CFU) treatment for 6 days, to evaluate DPC‐SFPL's effect on intestinal microbiota. Fecal samples were collected on day 10 for 16S rDNA sequencing. DSS disrupted gut flora structure, whereas DPC‐SFPL modulated and restored flora homeostasis. Principal component analysis (PCA) showed a clear separation of the healthy, DSS, and DPC‐SFPL groups (Figure 7a), confirmed using NMDS (Figure 7b), indicating that DPC‐SFPL affects community structure by influencing key flora. Compared with the DSS model group, DPC‐SFPL significantly increased the abundance of *Lactobacillus* species and suppressed pathogenic bacterial genera (Figure 7c), altering microbial abundance at the phylum and order levels (Figure 7d, e). A heatmap of the top 30 phyla showed significant compositional differences between the groups (Figure S28a). The phylogenetic tree revealed taxonomic differences among the groups, with the significant taxa highlighted (Figure S28b). Quantitative analysis showed that *Lactobacillus* was reduced in DSS mice but was restored after DPC‐SFPL treatment (Figure 7f–o). Overall, DPC‐SFPL partially reversed DSS‐induced dysbiosis by inhibiting proinflammatory taxa, re‐establishing barrier‐associated taxa, and restoring metabolically functional taxa.

## 3 Discussion

This study describes a dual‐key genetic circuit in *EcN* that achieves antibiotic‐free plasmid stability and self‐regulated lysis for therapeutic protein delivery. By deleting both *asd* and *thyA*, bacterial survival strictly depended on dual‐plasmid complementation, ensuring stable gene expression and biosafety. The quorum‐sensing lysis module enables population control and periodic release of functional proteins [16, 23], while the expression module co‐delivers an IL‐2 mutant and Amuc_1100 to restore immune balance and intestinal barrier integrity [47]. This design effectively alleviated colitis in mice by promoting Treg expansion, suppressing inflammation, and repairing epithelial junctions, accompanied by beneficial modulation of the gut microbiota [48, 49, 50]. Compared with traditional systems relying on antibiotics or single‐gene complementation, the dual‐key circuit offers superior stability, controllability, and therapeutic precision.

Previous studies have demonstrated the therapeutic potential of engineered bacteria for IBD. Multiple strategies have been developed, such as utilizing engineered *Escherichia coli* Nissle 1917 for oral protein delivery therapy [10], using bacteria responsive to inflammatory markers to monitor disease progression [49, 50] and coating bacteria with chitosan/sodium alginate to express molecules that scavenge reactive oxygen species and repair the intestinal barrier [49, 50]. Additionally, platforms have been constructed to secrete anti‐TNF‐α nanobodies for anti‐inflammatory effects or express repair factors to alleviate inflammation [9, 28, 29]. However, current strategies primarily focus on a single dimension, either emphasizing immune modulation or concentrating on barrier repair, lacking simultaneous intervention for both immune and barrier restoration. This study achieved a synergistic therapy by simultaneously delivering IL‐2 mutants and Amuc_1100 to regulate the immune response and promote barrier repair. The survival dependency and periodic lysis of the dual‐key circuit ensure stable delivery of functional proteins while preventing excessive population growth. This capability is absent in the existing engineered bacterial therapeutic systems.

Despite these advantages, dual‐plasmid deletion systems have some limitations. The treatment duration in the mouse IBD model was approximately 10 days, which is insufficient for the long‐term monitoring of plasmid retention efficiency and the therapeutic efficacy of the engineered bacteria. Future studies should use long‐term treatment models applicable to clinically relevant chronic diseases to better demonstrate the advantages of this system.

Overall, the modular design and enteric‐coated protection of this system ensured a targeted colonic release and sustained efficacy. Beyond UC, this platform also provides a flexible vehicle for the programmable delivery of diverse therapeutic payloads, including immunomodulators and metabolic enzymes. This establishes a safe, modular, and self‐regulating bacterial system that advances the development of next‐generation biotherapeutics for the treatment of immune and mucosal diseases.

## 4 Experimental Section

### 4.1 Cell Lines, Plasmids, and Animals

*EcN* (purchased from Bio SCI Company) were cultured at 37°C in LB broth with vigorous shaking. Then, *EcN*s were stored in a bacterial cryopreservation solution (50% LB broth, 25% sterile water, and 25% glycerol) at −80°C for further experiments. Caco‐2 cells (American Type Culture Collection, Rockville, MD) were provided by the China Standard Strain Preservation and Management Center and cultured in Dulbecco's Modified Eagle Medium (DMEM) containing 20% fetal bovine serum (FBS), 100 U mL^−1^ penicillin, and 100 µg mL^−1^ streptomycin in a humidified incubator at 37°C with 5% CO_2_. Cells were subjected to starvation treatment with RPMI 1640 medium containing 1% FBS for 24 h. Mixed proinflammatory factors (such as 10 ng mL^−1^ IL‐1β + 20 ng mL^−1^ TNF‐α + 20 ng/mL IFN‐γ) were added to the lower side of the Transwell chamber to stimulate cells and induce inflammatory damage to the intestinal epithelial barrier.

Female BALB/c mice (6–8 weeks) were purchased from Jiangsu Jicui Pharmachem Biotechnology Company. Mice were housed in individually ventilated cages with 5 mice per cage, which were regularly maintained on a 12 h:12 h light:dark cycle (8 a.m. to 8 p.m.; darkness at night) with controlled temperature (21 ± 1°C) and humidity (40%–70%). Food and water were provided ad libitum. All animal experiments were approved by the Institute of Health and Medicine (IHM), Hefei Comprehensive National Science Center Animal Ethics Committee (Approval No. IHM‐AP‐2024‐062‐R1).

### 4.2 Construction and Characterization of Engineering Bacteria

*EcN* was used as the chassis strain, and the *asd* and *thyA* genes on the chromosome were knocked out by the λ‐Red system to construct a double auxotrophic strain of *EcN^ΔasdΔthyA^*. pKD46 was electro‐transferred to *EcN*, and the expression of pKD46 was induced by L‐(+)‐arabinose and prepared in an electro‐transferred sensory state after several washes. The specific steps for this experiment were as follows: the chloramphenicol‐resistant PKD3 fragment was amplified using pKD3 forward and pKD3 reverse primers. Homologous arms of the *asd* gene (>50 bp) were added to both sides of the pKD3 primers (pKD3‐*asd*‐Forward and pKD3‐*asd*‐Reverse). Primers with homologous arms were used to amplify the pKD3 sequence, and the resistant fragments carrying the homologous arms were recovered following agarose gel electrophoresis. The recovered fragments were simultaneously transferred to L‐(+)‐arabinose–induced pKD46‐*EcN* competent cells, plated on chloramphenicol‐resistant plates supplemented with DAP, and validated for knockout efficiency via colony PCR and Sanger sequencing. Subsequently, the cells were transferred to a PCP20 plasmid to eliminate resistance genes. *thyA* was knocked out using pKD4; all other procedures were identical. The primers used are listed in Table S1.

A bifunctional expression plasmid was constructed via seamless cloning by placing the fluorescent reporter GFP downstream of the constitutive promoter J23119 and mCherry downstream of tac to generate independent plasmids. Subsequently, they were seamlessly cloned into a vector containing the p15a replication origin and the chloramphenicol resistance gene, and a plasmid expressing both GFP and mCherry was constructed. For the expression protein plasmid, GFP was replaced with the Flag‐tagged IL‐2 mutant N103R/V106D, mCherry with His‐tagged Amuc_1100, and the *thyA* gene from the *EcN* genome was inserted as a thymidine synthesis compensatory module.

The synthesized lysis plasmid was constructed with pTD103luxI‐sfGFP as the backbone (pTD103luxI_sfGFP), which was a gift from Jeff Hasty (Addgene plasmid #48885; http://n2t.net/addgene:48885). In this construct, the sfGFP gene, was replaced with the phage *ϕX174E* lysis gene E, whose expression was regulated by the pLux promoter. The plasmid also carried a kanamycin resistance marker and an inserted *asd* gene to compensate for defective DAP synthesis in the host bacterium. The primers used were listed in Table S1.

The expression and lysis plasmid were introduced into the *EcN^ΔasdΔthyAΔpMUT^* host strain by electroporation (1.8 kV, 5 ms), and positive clones of the transformed strain were screened in antibiotic‐free LB medium.

### 4.3 Western Blot

Total bacterial and supernatant proteins were collected using the Bacterial Active Protein Extraction Kit, according to a time gradient, and then separated using sodium dodecyl sulfate‐polyacrylamide gel electrophoresis (SDS‐PAGE). After protein transfer, the polyvinylidene fluoride (PVDF) membrane was blocked with 5% bovine serum albumin (BSA) in TBST, and then incubated overnight at 4°C with anti‐Flag or anti‐His horseradish peroxidase (HRP)‐conjugated antibodies. The PVDF membranes were washed with TBST and exposed to enhanced chemiluminescence.

### 4.4 Therapeutic Effect on the UC Model

Thirty female BALB/c mice (6–8 weeks) were divided into five groups of six mice each and acclimatized for 1 week before further experiments. For the DSS‐induced mouse UC model, mice were fed drinking water containing 3% DSS for 5 days and then replaced with plain water. Healthy mice in the control group were administered plain water. Treatment was performed using SL, SFPL, and DPC‐SFPL by gavage at a concentration of 1 × 10^8^ CFU/mouse. The mouse weights were recorded daily during the treatment period. At the end of the treatment, the mice were sacrificed, and their distal colon tissues were collected for different evaluations. Then, to verify the beneficial effects of intestinal flora during UC treatment, mice were administered a mixed antibiotic solution containing 1 g L^−1^ metronidazole, 1 g L^−1^ neomycin, 0.5 g L^−1^ vancomycin, and 1 g L^−1^ ampicillin for 5 consecutive days, followed by 3% DSS treatment. During the treatment period, the body weights of the mice were recorded daily. At the end of treatment, the mice were sacrificed, and their distal colon tissues were collected for different evaluations, including H&E staining, colonic permeability assay, and fluorescent staining assay for colonic barrier proteins.

### 4.5 In Vivo Intestinal Integrity Assessment

Integrity of the intestine was evaluated using the FITC‐dextran assay. Briefly, female BALB/c mice (deprived of food and water for 4 h) were orally administered FITC‐dextran (4 kDa, 0.6 mg g^−1^) and serum was collected 3 h later to detect the FITC fluorescence signal.

### 4.6 Analysis of Disease Activity

The viscosity and presence of fecal blood were observed daily during UC treatment in mice from different treatment groups. Parameters representing the DAI were recorded, as shown in Table S2, and their corresponding scores were obtained from previous publications. The DAI was obtained by summing the scores of different parameters.

### 4.7 Histological Analysis

The distal colons of mice were collected from mice and fixed with 4% paraformaldehyde for 24 h before H&E staining. The colon tissue was embedded in paraffin, sectioned for H&E staining, and observed under a light microscope. Colonic tissue injury was assessed using a blinded method according to previously described criteria to avoid observer bias. The colonic epithelial damage and inflammatory cell infiltration scores are presented in Table S3.

### 4.8 MPO Activity Assay

To detect MPO activity, mouse colon tissues were collected after treatment and digested with collagenase IV and hyaluronidase (grade I). MPO activity in the colon tissue was assayed using an MPO Activity Assay Kit. Manufacturer's instructions were not provided.

### 4.9 Colonic Tissue Cytokine Assay

To detect the levels of inflammatory cytokines, colon tissues were collected from mice. Fresh colon tissues were rinsed with pre‐cooled PBS, weighed, and sheared into approximately 1 mm^3^ tissue blocks, which were added to pre‐cooled RIPA lysate (containing 1% protease inhibitor mixture) at a mass‐to‐volume ratio of 1:9, and mechanically homogenized on ice until the tissues were completely fragmented. The homogenate was allowed to stand for 30 min at 4°C, and then centrifuged at 12 000 × *g* for 20 min (4°C), and the supernatant was collected and stored in separate fractions at −80°C for backup and to detect cleavage protein tag protein. Total protein concentration was determined via the BCA method with BSA as the standard, and all samples were diluted to fall within the linear range of the assay (20–2000 µg mL^−1^). The final cytokine concentrations (assessed using ELISA) were normalized to the total protein concentration. ELISA assays for TNF‐α, IL‐6, IL‐10, and TGF‐β were performed following the manufacturer's instructions.

### 4.10 In Vivo Immunofluorescence Staining Analysis

After treatment, mouse colon tissues were collected and fixed in 4% paraformaldehyde (4°C, 24 h). The tissues were then sequentially dehydrated through gradient ethanol, cleared in xylene and embedded in paraffin. Paraffin sections (4 µm thick) were prepared and baked at 60°C for 2 h to enhance tissue adhesion. After dewaxing and rehydration, the sections underwent heat‐induced antigen retrieval in sodium citrate buffer (pH 6.0) at 95°C for 20 min to expose the antigenic epitopes. The sections were then permeabilized with 0.1% Triton X‐100 for 10 min and blocked with 5% BSA for 1 h (room temperature) to prevent nonspecific binding. Primary antibodies (rabbit anti‐Occludin, 1:200; mouse anti‐ZO‐1, 1:200) were incubated overnight at 4°C. After washing with PBS, sections were incubated with fluorescent secondary antibodies (Alexa Fluor 488 anti‐mouse and Alexa Fluor 647 anti‐rabbit, 1:500) for 1 h in the dark. Nuclei were counterstained with DAPI. Negative controls were performed using PBS instead of the primary antibody, and single‐label and isotype controls were included to exclude nonspecific signals. Images were acquired using confocal microscopy, and ZO‐1, Occludin, and nuclei were detected at 488, 647, and 405 nm, respectively.

### 4.11 In Vivo Toxicity Assessment

Ten female BALB/c mice (6–8 weeks) were divided into two groups (five mice per group) and orally administered PBS or DPC‐SFPL on days 3, 5, and 7. The body weights of mice were measured daily. The mice were euthanized on day 10, and blood samples were collected and sent to Anhui KETU Biotechnology Co., Ltd. for routine blood and blood biochemical evaluation. The major organs were collected for histopathological evaluation using H&E staining.

### 4.12 Statistics Analysis

The data are expressed as the mean ± SEM. Statistical differences between the control and experimental groups were analyzed using one‐way analysis of variance (ANOVA) with Tukey's post hoc test. Paired samples were compared via paired *t*‐tests. Statistical analyses were performed using GraphPad Prism 5. The sample sizes for each study are provided in the Figure legends. These data were not excluded from the analyses. Statistical significance is indicated as *p < 0.05, **p < 0.01, ***p < 0.001, and ****p < 0.0001.

## Author Contributions

**W.X**. and **W.F.D**. conceived and designed the study, supervised the project, and wrote the manuscript. **S.J.D**. and **B.Y**. designed and performed most of the experiments and conducted data analyses. **S.J.D**. and **B.Y**. performed the immunophenotypic analysis of lymphocytes. **W.X**. and **S.J.D**. wrote and edited the manuscript, analyzed the data, and generated graphs. **W.X**. and **W.F.D**. oversaw the project and study design and secured funding to support the research.

## Conflicts of Interest

The authors declare no conflicts of interest.

## Supporting information




**Supporting File**: advs74400‐sup‐0001‐SuppMat.docx.

## Data Availability

The primary supporting data for the results of this study are included in the manuscript and supplementary information. The raw and analyzed datasets generated during the course of this study are available for scientific use upon reasonable request from the corresponding author. The source data for the figures and tables are provided in this paper.
