# Supplementary material for: Dual‐Key Genetic Circuit Enables Stable and Self‐Regulated Engineered Bacteria for the Treatment of Ulcerative Colitis
Source: Adv Sci (Weinh). 2026 Feb 13;13(23):e20434. doi: 10.1002/advs.202520434 (PMC13104102; doi:10.1002/advs.202520434)
Supplement: Supplementary file 1 — Supporting File: advs74400‐sup‐0001‐SuppMat.docx. [file ADVS-13-e20434-s001.docx]

**Supporting Information**

**Dual-Key Genetic Circuit Enables Stable and Self-Regulated Engineered Bacteria for the Treatment of Ulcerative Colitis**

Shuaijie Ding ^1, 4^, Biao Yang ^1, 4^, Xinyu Li^1, 4^, Yi Wang^1^, Chunyu Huang^2^, Wenfei Dong, ^3, *^ Wei Xie^1^^, *^

^1^ Institute of Health and Medicine (IHM), Hefei Comprehensive National Science Center, Hefei, Anhui, 230000, China.

^2^ Department of Oncology, Tongji Hospital, Tongji Medical College, Huazhong University of Science and Technology, Wuhan, 430030, Hubei, China.

^3^ Suzhou Institute of Biomedical Engineering and Technology, Chinese Academy of Science (CAS), Suzhou 215163, China.

^4^ These authors contributed equally to this study.

* e-mail: [xiew@ihm.ac.cn](mailto:xiew@ihm.ac.cn), wenfeidong@sibet.ac.cn

**Keywords:** Engineered bacteria; Ulcerative colitis; Genetic circuit; Quorum sensing;

**This PDF file includes:**

Supplementary Text

Figs. S1 to S28

Tables S1 to S5

**Supplementary Text**

**Experimental Section**

*Construction of cryptic plasmid-cured EcN: EcN* cryptic plasmids were cured with Cas9-mediated double-strand break, Briefly, *EcN* was transformed with pFREE or pCryptDel4.8 to cure the cryptic plasmids pMUT1 or pMUT2, respectively. The transformants were grown overnight and diluted 1:1000 the next day into fresh LB containing 0.2 % rhamnose and 0.43 μM anhydrotetracycline. After 24 h of incubation, the culture was streaked onto LB plates without antibiotics and incubated overnight in a 30 ℃ incubator. Colonies were screened with colony PCR to verify the loss of cryptic plasmids.

*Plasmid retention rate detection:* Plasmid retention frequency was estimated by the proportion of plasmid-containing cells in selective medium after overnight culture. For plasmid loss frequency, single colonies from selective medium (ensuring plasmid carriage) were inoculated, cultured at 37 ℃ for 12 h, then serially diluted and plated on non-selective medium. Colonies from non-selective medium were transferred to both non-selective and selective plates, the ratio of colonies on selective to non-selective plates represented the proportion of plasmid-containing cells.

*Preparation and characterization of EcN-L100:* The preparation of *EcN*-L100 was carried out according to previous reports. *EcN* was dispersed in a calcium chloride solution (12.5 mM) in an ice bath. Then, an L100 solution was added and further vortexed for 5 min. the concentration of L100 in the mixed solution was 0.04 mg/mL and the pH was adjusted to 5.0 Then, the *EcN*-L100 was washed with PBS and centrifuged for collection. To assess the formation of L100 coating on the *EcN*-GFP surface, Cy5.5 was labeled with L100, and the fluorescence intensity of *EcN*-GFP with or without L100-Cy5.5 coating was assessed by flow cytometry. As well as the bacterial fluids with or without L100-Cy5.5 coated *EcN*-GFP were dropwise added to slides, respectively, and the slides were sealed after drying away from light. Images were acquired using a laser confocal microscope, Cy5.5 channel and GFP channel.

*In vitro and in vivo resistance analysis of EcN-L100: EcN* with or without L100 coating was resuspended in PBS (control), SGF, at 37 °C with gentle shaking for 2 h. Bacteria were collected from each sample by centrifugation at different time points, diluted by washing with PBS, and 100 μL of each diluted sample was applied to solid agar plates containing kanamycin and chloramphenicol. The number of bacteria was determined by the number of colonies after 24 h of incubation in a microbiological incubator. To further determine the survival of probiotics in the GI tract, female Balb/c mice were orally administered *EcN*-Lux or *EcN*-Lux-L100 (1 × 10^8^/CFU), and then the mice and their gastrointestinal tracts were imaged with an *in vivo* IVIS imaging system (IVIS, Luminescence III). Bioluminescent signals in the region of interest were quantified by IVIS image 4.2 software and expressed as per second/cm^2^ (p/s/cm^2^/sr).

*Flow cytometry analysis:* Colonic lamina propria samples from different groups of mice were obtained and homogenized using a tissue grinder, resulting in the formation of cell suspensions. The obtained suspensions were washed twice with cold PBS and stained with fluorescent staining using mouse monoclonal antibodies conjugated including APC/Cy7-CD45, PE-CD11b, FITC-Ly6G and Percp/Cy5.5-CD4, and APC-CD25 for 30 min at room temperature. For intracellular staining, cells were fixed in 4 % paraformaldehyde (PFA) for 30 min at room temperature. After washing with Perm/wash buffer, the stained cells were incubated with PE-Foxp3 and antibodies for 15 min at room temperature and analyzed using a BD flow cytometer and FlowJo software.

*Gene expression analysis by qRT-PCR:* RNA from stable tissue samples was eluted with 100 µL kit-provided RNase-free water. Colon RNA extraction required a strict RNase-free environment to preserve RNA integrity and purity. Prior to experimentation, liquid nitrogen and pre-cooled PBS with 1 % RNase inhibitor were prepared. Following animal sacrifice, 50-100 mg colon tissue was rapidly dissected, with intestinal lumen contents gently rinsed three times in pre-cooled PBS to prevent mechanical damage. Tissues were then cut into 5 mm³ pieces, quick-frozen in liquid nitrogen, stored at -80 ℃, ground in liquid nitrogen, rinsed with pre-cooled PBS containing 0.1 % DEPC water to remove non-target components, and total RNA extracted with TRIZOL. RNA concentration was measured with a Nanodrop. For each target gene (Supplementary Table 4), 10 ng RNA was analyzed with specific primers using a one-step qRT-PCR kit and Real-Time PCR Detection System per manufacturer’s instructions. Expression results were normalized using the 2^-ΔΔCt^ method. Expression of each gene was normalized to that of housekeeping gene glyceraldehyde-3-phosphate dehydrogenase (GAPDH).

*Gut microbiota analysis by 16S sequencing:* After different treatments, mouse feces were collected, frozen in liquid nitrogen, and sent to Genetic Research for gut flora analysis by 16S sequencing. Specifically, microbiome DNA was sequenced by the Magen Hipure Soil DNA Kit (Magen, Cat. A 16S rRNA library was constructed without utilizing the VAHTS Universal DNA Library Preparation Kit. On an Illumina model (Illumina, Novaseq 6000). It was constructed using genewiz's proprietary primers "forward primer" and "reverse primer", which target the V3 and V4 highly variable regions of microbiota 16S rDNA. 16S was analyzed by rRNA gene sequencing using the QIIME 2 data analysis package. Specifically, forward and reverse sequences were assigned to the samples based on barcodes, followed by further removal of barcodes and primer sequences. The resulting products were filtered to remove sequences that contained ambiguous bases, were longer than 200 bp, or had an average mass fraction of less than 20. Chimeric sequences were identified by a reference database (RDP Gold database) and the UCHIME algorithm and discarded to obtain valid sequences for final analysis. Sequences were clustered into operational taxonomic units (OTUs) using the clustering program VSEARCH (1.9.6) with 97% sequence homology. the 16S rRNA reference database was Silva 132, and taxonomic category analysis was performed on all OTUs using the Ribosomal Database Program (RDP) classifier with a confidence threshold of 80 %. Finally, based on the OTU analysis results, the Shannon index was calculated in the QIIME 2 data analysis package


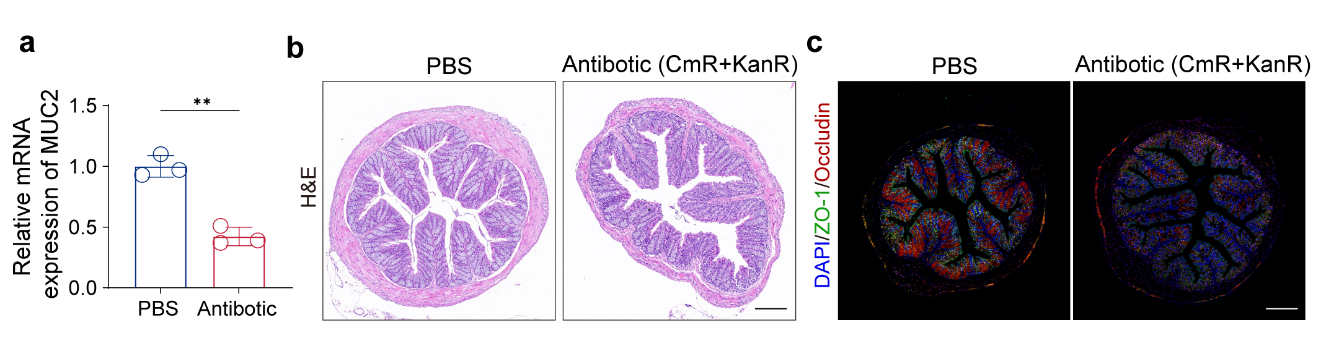


**Fig. S1.** Antibiotic treatment was followed by extraction of RNA from the proximal colon tissue of mice. The relative expression levels of MUC2 in the colon were detected by qPCR **(a)**, and HE staining of the colon was performed **(b)**. Data are presented as mean values ± SEM (n = 3 biologically independent samples). Statistical significance was calculated compared with the PBS group. P values determined by Student’s two-sided t-test. (**P* < 0.05, ***P* < 0.01, ****P* < 0.001 and *****P*<0.0001).


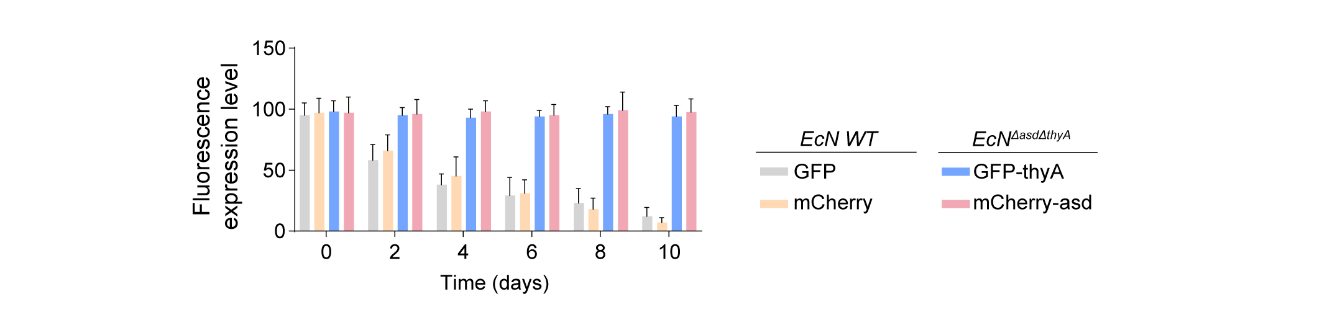


**Fig. S2.** *EcN* wild-type and *EcN^ΔasdΔthyA^* knockout strains simultaneously expressing GFP and mCherry, respectively. The knockout strain carries *asd* and *thyA* compensation. consecutive passages without antibiotic, fluorescence intensity detected.


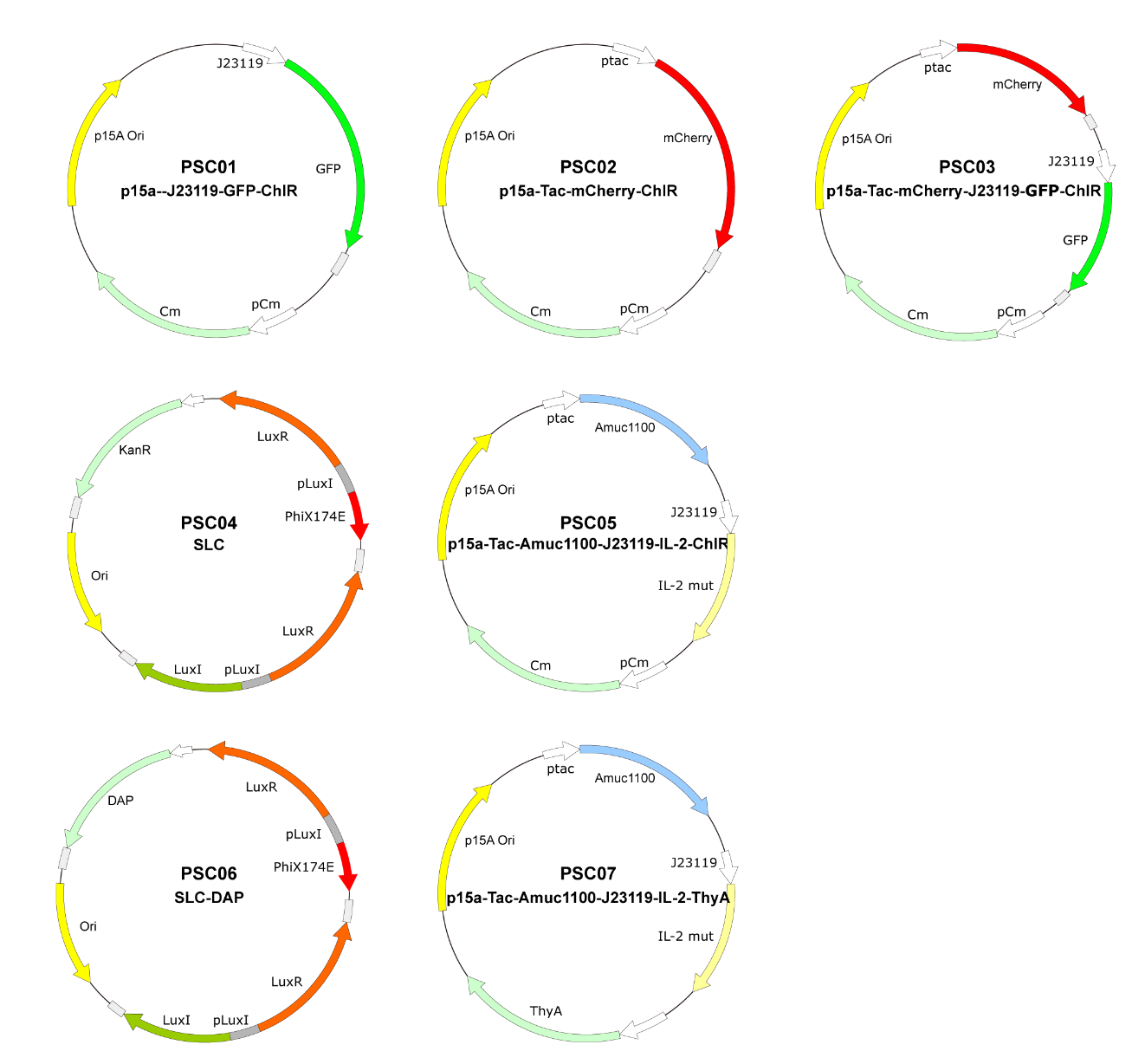
 **Fig. S3.** The main plasmids used in this study.


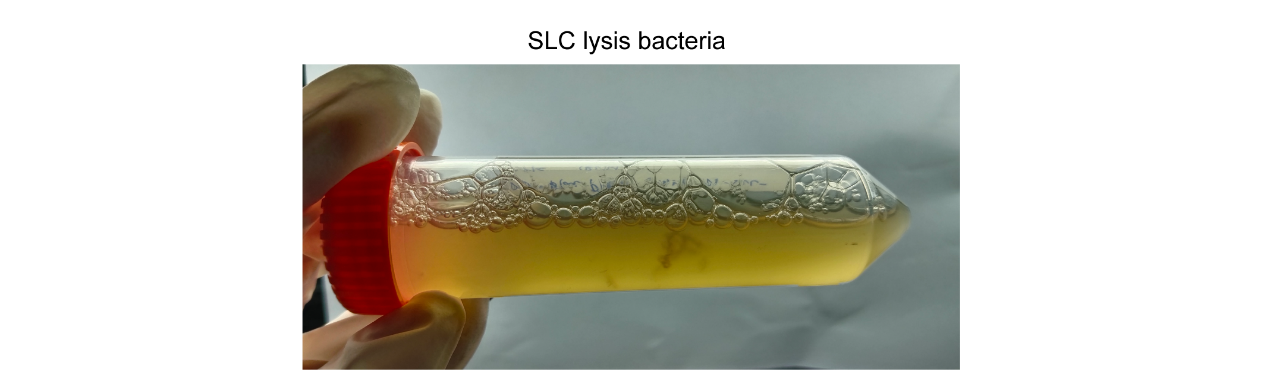


**Fig. S4.** Pictures of flocculation occurring during the lysis of SLC engineered bacteria in LB continuous culture.


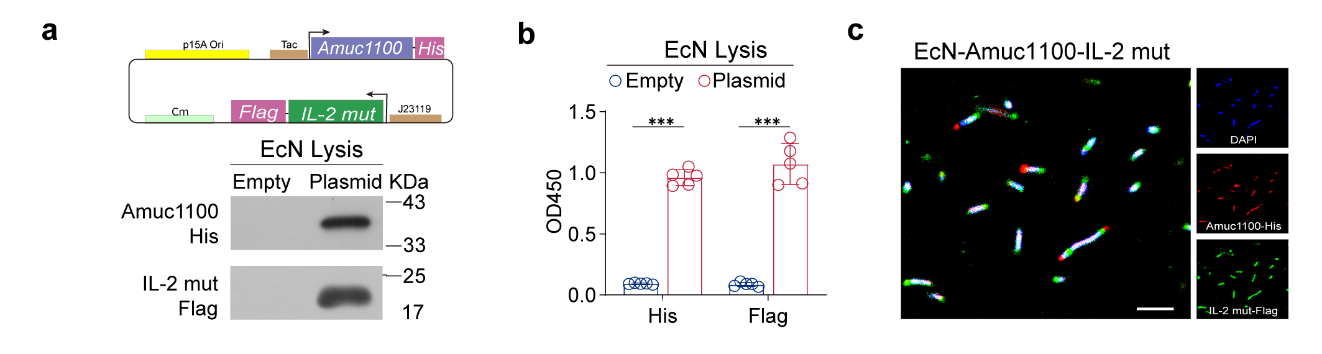
 **Fig. S5. a-b** Westen Blot **(a)** and Elisa **(b)** detection of Amuc_1100 and IL-2 mutant expression. **c** immunofluorescence detection of Amuc_1100 and IL-2 protein expression in strains, confocal detection of his tag (red) and Flag tag (green). Amuc_1100 has a his tag, IL-2 has a flag tag, and DAPI stains bacterial morphology. Scale bar: 2 μm. Data are presented as mean values ± SEM (n = 5 biologically independent samples). Statistical significance was calculated compared with the PBS group. P values determined by by Student’s two-sided t-test. (**P* < 0.05, ***P* < 0.01, ****P* < 0.001 and *****P*<0.0001).


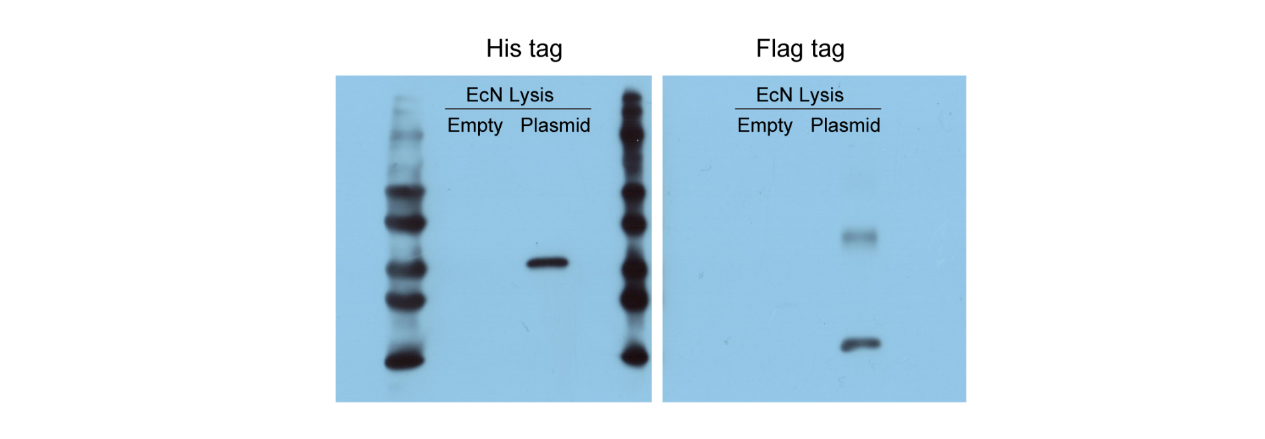


**Fig. S6.** Raw images of Western Blot detection for Amuc_1100 and IL-2 mutant expression


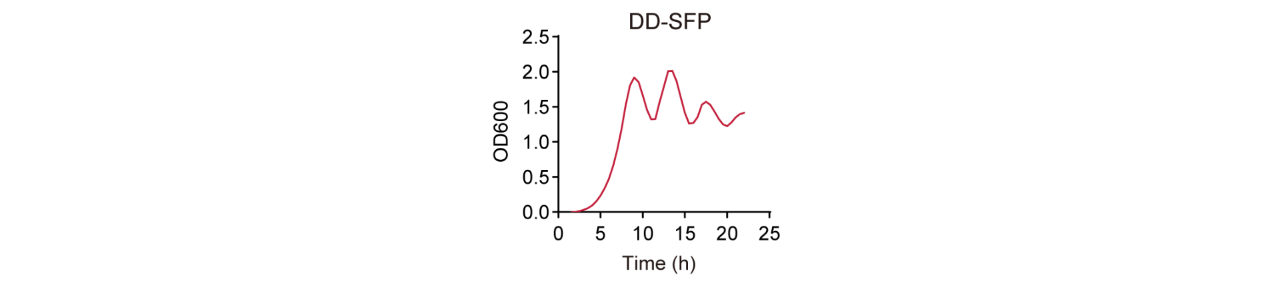


**Fig. S7.** Images showing continuous monitoring of bacterial growth concentration using dual-plasmid compensation with SLC and functional proteins to overcome host gene defects.


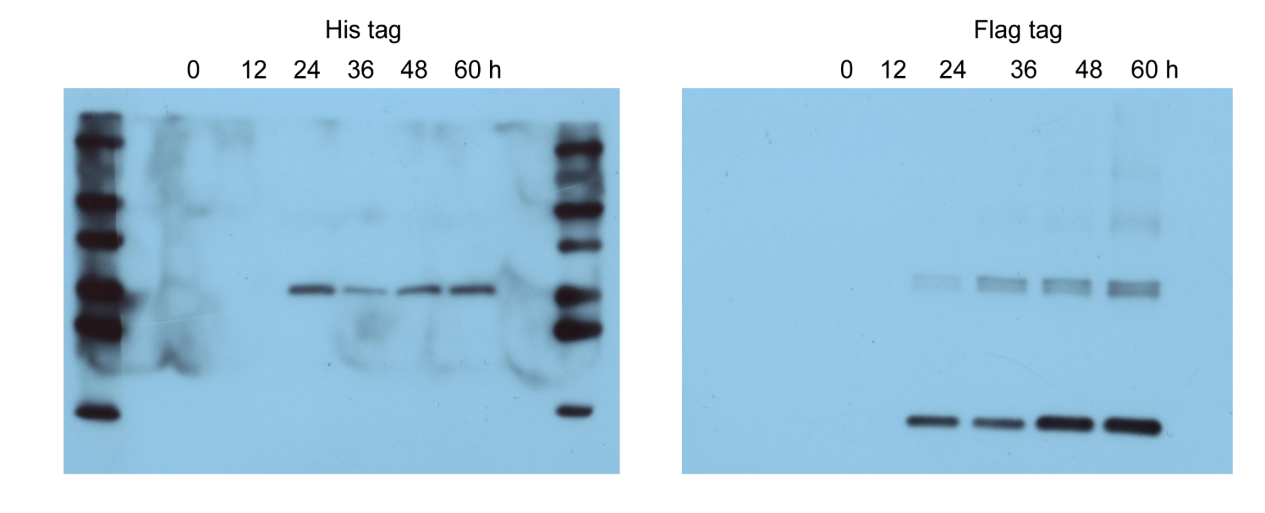
 **Fig. S8.** Collect DPC-SPF supernatant at time points, Westen Blot detection of Amuc_1100 and IL-2 mutant expression raw images


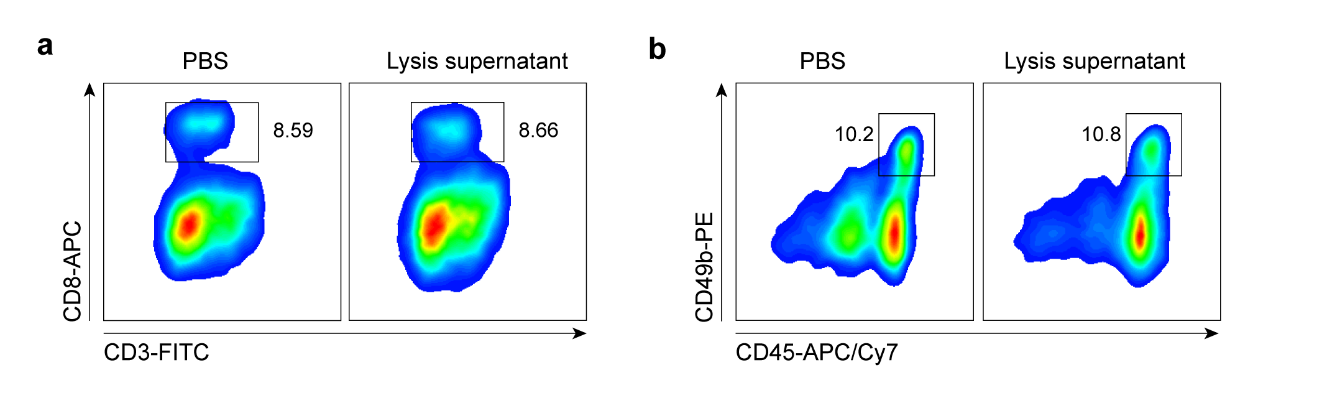


**Fig. S9. a-b** PBS or bacterial lysate supernatant was intravenously administered to mice, and CD8^+^ T cells (CD3^+^CD8^+^) **(a)** and NK cells (CD45^+^CD49b^+^) **(b)** in the spleen tissue were analyzed by flow cytometry.


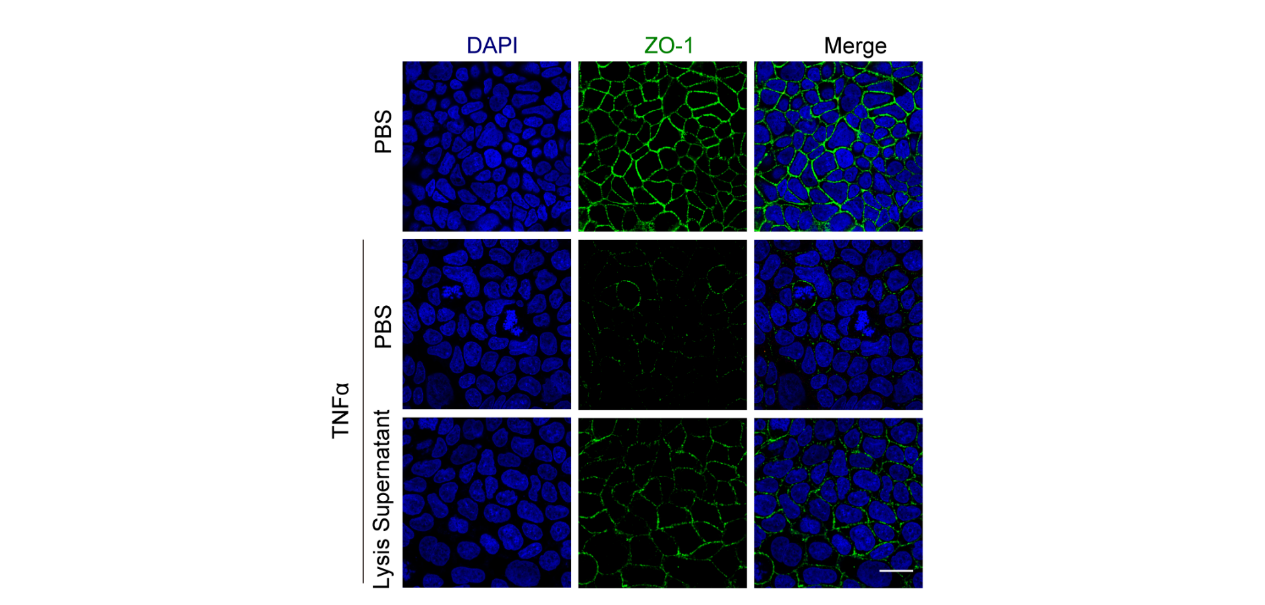


**Fig. S10.** Immunofluorescence detection of ZO-1 expression in Caco-2 damage cell models treated with lysate supernatant. green (ZO-1) and blue (DAPI). Scale bar, 10 μm.


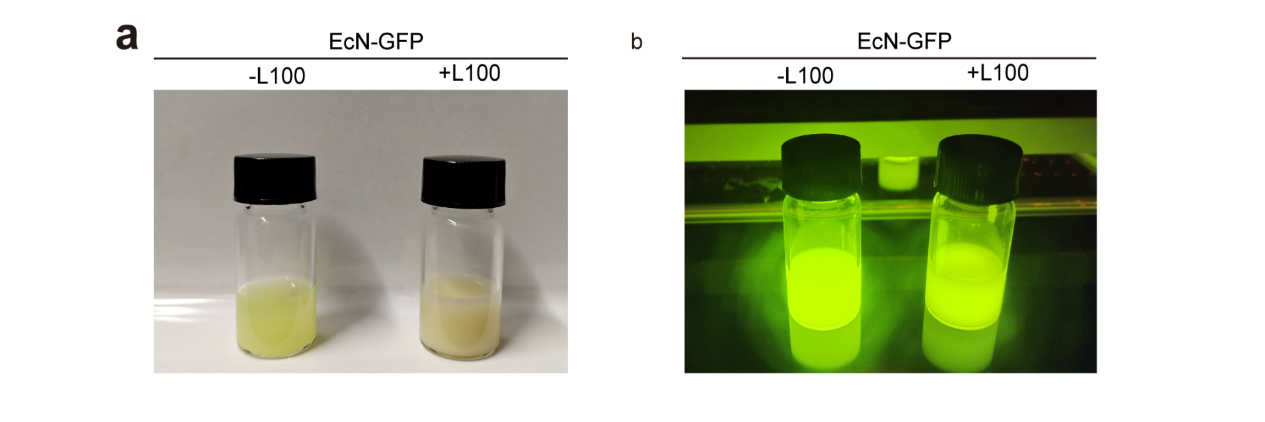


**Fig. S11. a** Images of *EcN*-GFP strain with and without L100-55 enveloping. **b** Images of *EcN*-GFP strain with and without L100-55 encapsulation under 488 nm excitation light.


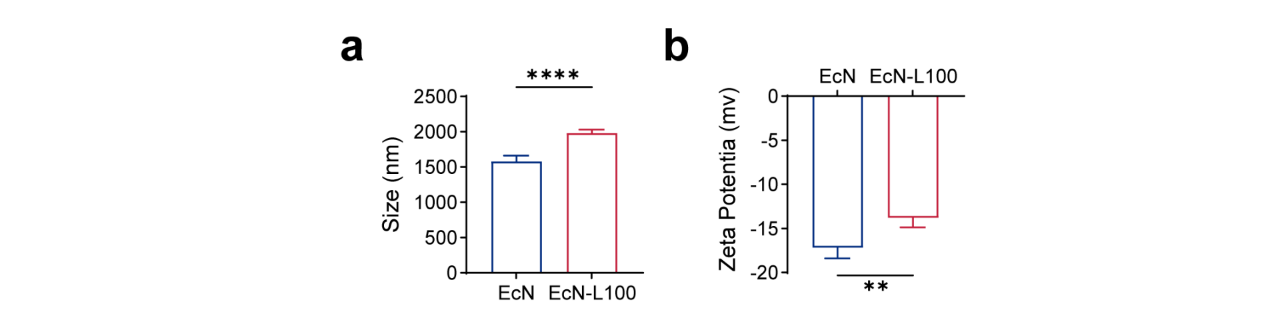


**Fig. S12.** L100-55 encapsulation *EcN*, strain size **(a)** and zeta potential **(b)** analysis


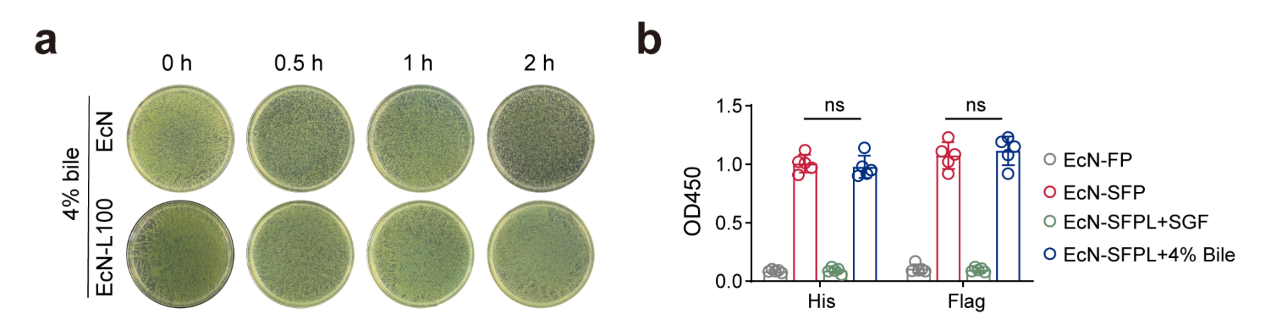


**Fig. S13. a** Bacteria were collected at fixed time intervals and spread onto resistance agar plates. Resistance plate images of *EcN* and *EcN*-L100 strains were obtained in a 4 % bile environment. **b** Content of His and Flag tags in ELISA-detected supernatants from different treated strains under SGF and 4 % bile conditions.


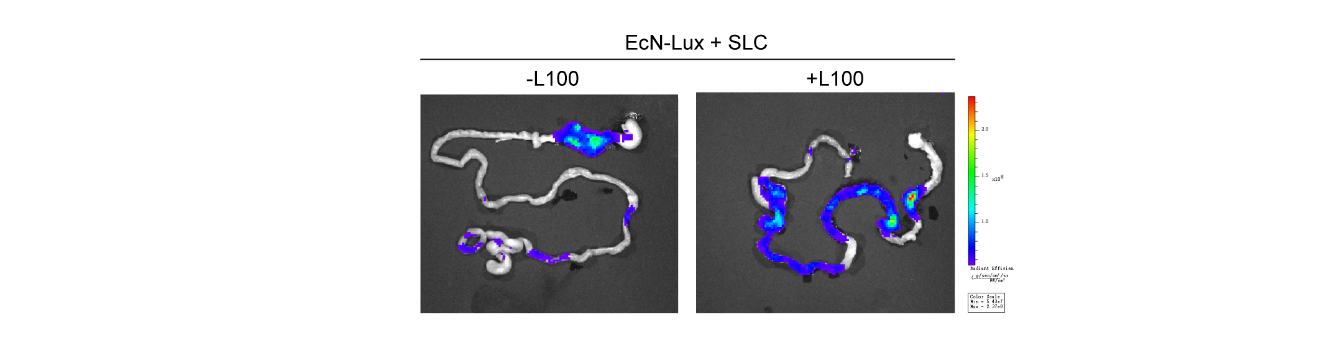


**Fig. S14.** Images of bioluminescence imaging at the proximal colon region in mice 24 hours following oral administration of L100-encapsulated *EcN*-Lux-SLC strain.


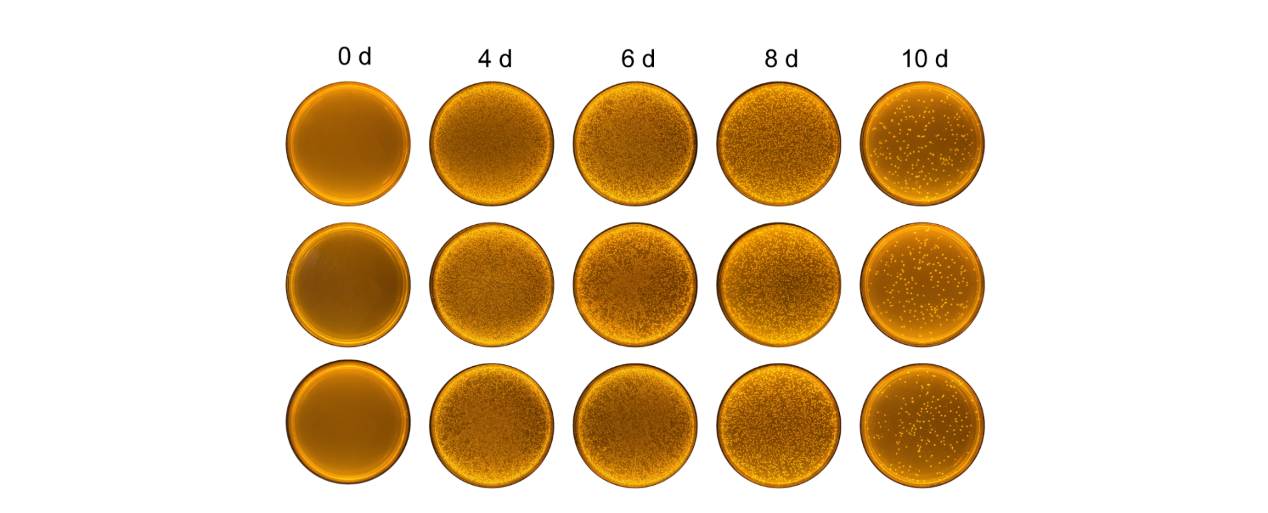


**Fig. S15.** DPC-SGL (1×10⁸ /CFU) was administered orally to mice three times, with a two-day interval between administrations (on days 3, 5, and 7). Mouse feces were collected at regular intervals (on days 0, 4, 6, 8, and 10)., diluted, and spread on resistant plates. The plates were incubated at 37 ℃ for 24 hours, and bacterial colonies were observed to determine the bacteria retained in the mouse intestine.


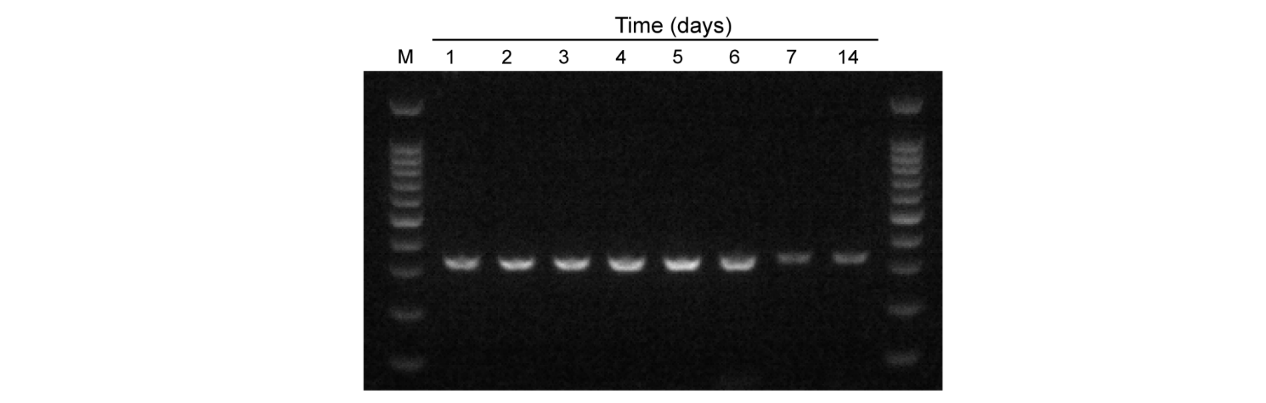


**Fig. S16.** Collect mouse feces periodically and measure the target gene fragment in the strain using PCR.


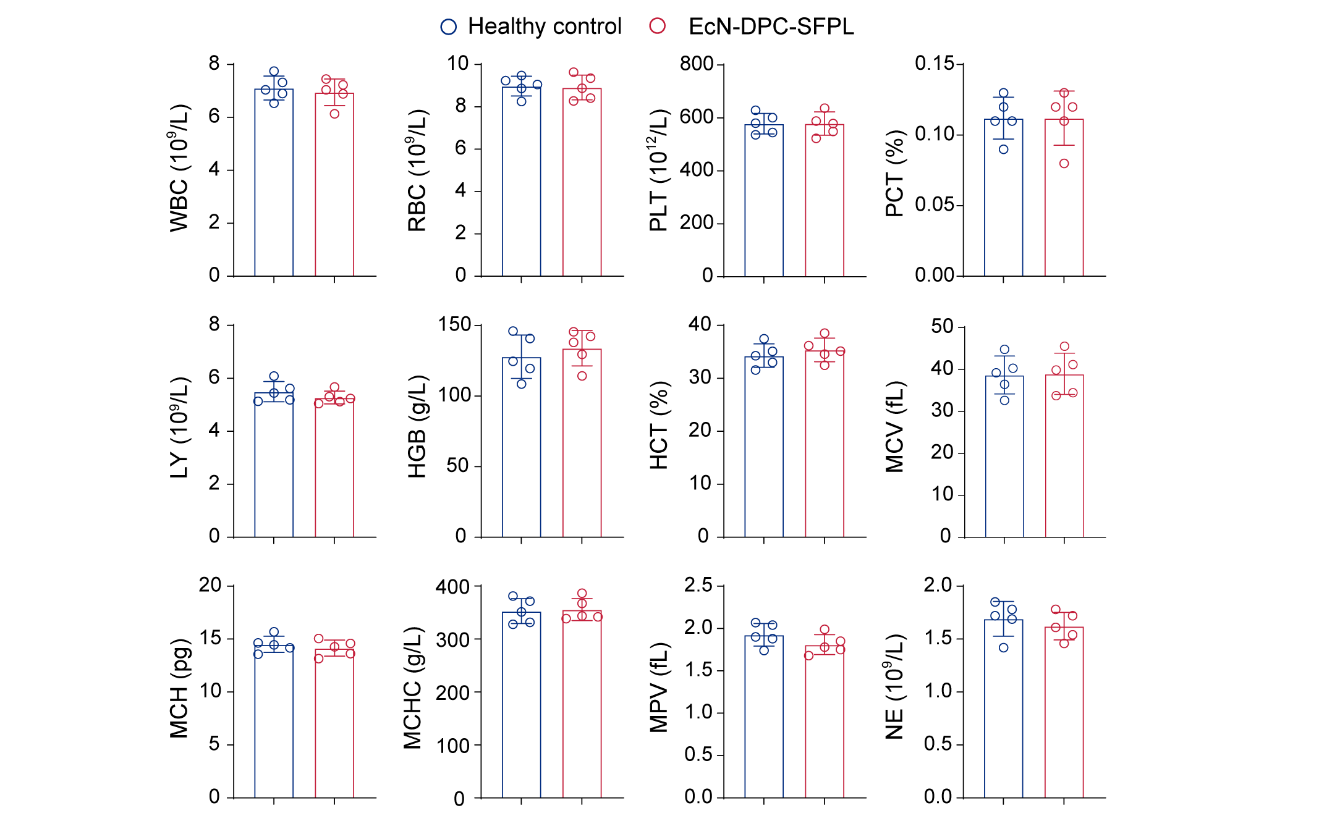
 **Fig. S17.** DPC-SFPL (1×10⁸ CFU) was administered orally to mice three times, with a two-day interval between administrations (on days 3, 5, and 7). On day 10, blood samples were collected from the mice for complete blood count analysis (n = 5 biologically independent samples). WBC:​​ White Blood Cell, RBC:​​ Red Blood Cell, PLT:​​ Platelet, PCT:​​ Plateletcrit, LY:​​ Lymphocyte, HGB:​​ Hemoglobin, HCT:​​ Hematocrit, MCV:​​ Mean Corpuscular Volume, MCH:​​ Mean Corpuscular Hemoglobin, MCHC:​​ Mean Corpuscular Hemoglobin Concentration, MPV:​​ Mean Platelet Volume, NE:​​ Neutrophil.


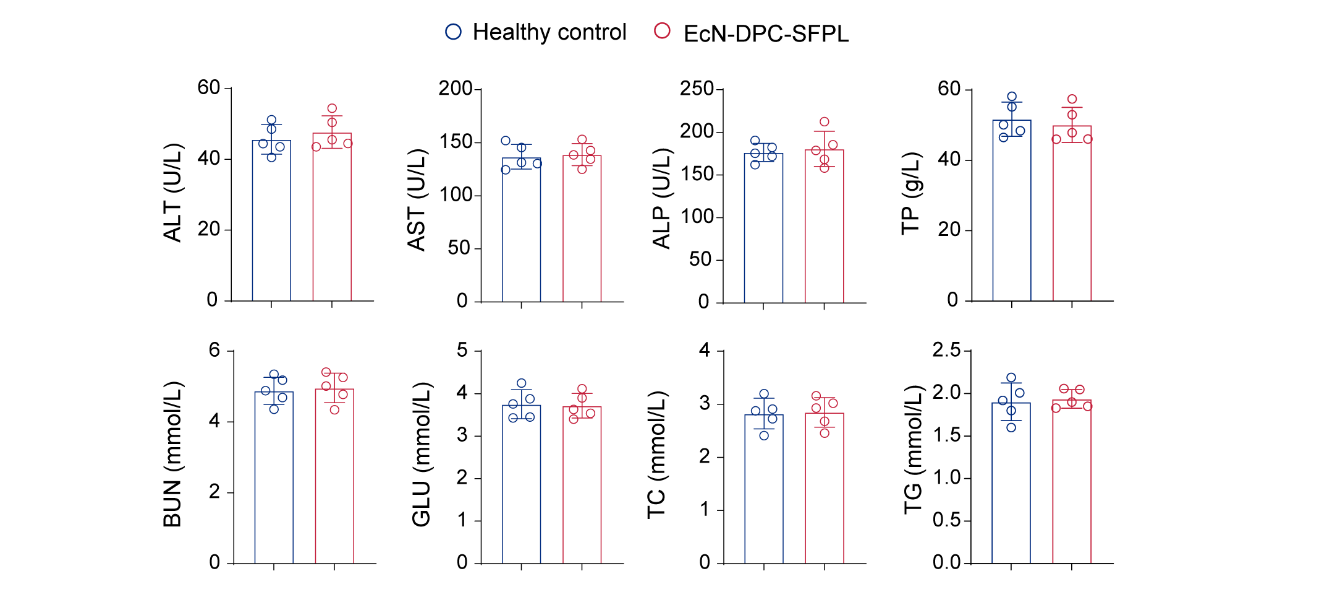


**Fig. S18.** DPC-SFPL (1×10⁸ CFU) was administered orally to mice three times, with a two-day interval between administrations (on days 3, 5, and 7). On day 10, blood samples were collected from the mice for complete blood biochemical analysis (n = 5 biologically independent samples). ALT:​​ Alanine Aminotransferase (U/L), AST:​​ Aspartate Aminotransferase (U/L), ALP:​​ Alkaline Phosphatase (U/L), ​TP:​​ Total Protein (g/L), ​BUN:​​ Blood Urea Nitrogen (mmol/L), GLU:​​ Glucose (mmol/L), ​TC:​​ Total Cholesterol (mmol/L), TG:​​ Triglycerides (mmol/L).


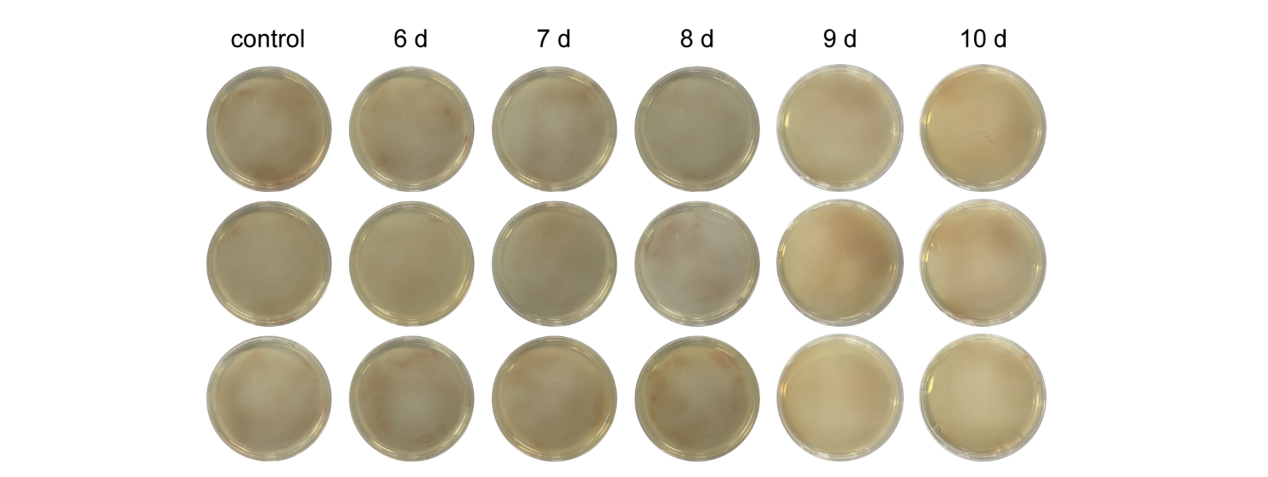


**Fig. S19.** DPC-SFPL (1×10⁸ CFU) was administered orally to mice three times, with a two-day interval between administrations (on days 3, 5, and 7). On day 6 to day10, blood samples were collected from the mice, and plate images of the spread plates were obtained (n=5 biologically independent samples).


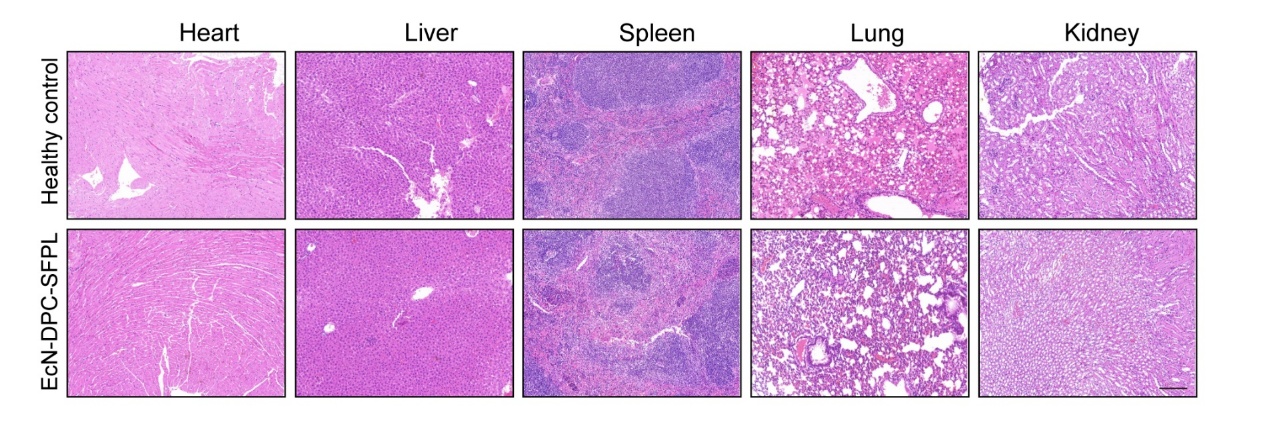


**Fig. S20.** HE-stained images of the heart, liver, spleen, lungs, and kidneys of mice were obtained (n = 5 biologically independent samples). Mice were administered DPC-SFPL (1×10⁸ CFU) orally three times, with a two-day interval between each administration (on days 3, 5, and 7). Scale bar: 100 μm.


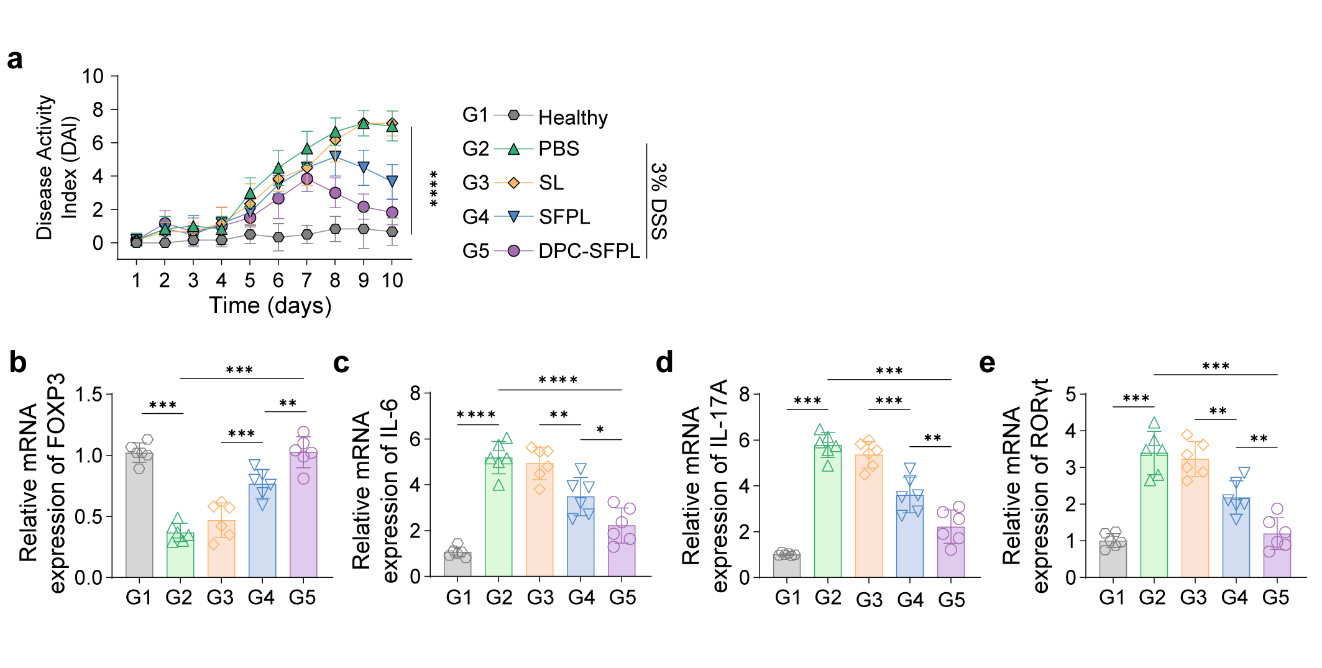
 **Fig. S21. a** Statistical graph of mouse DAI damage index at different weeks of engineered bacteria treatment. **b-d** Fluorescence quantitative PCR detection of relative gene expression of FOXP3 **(b)**, IL-6 **(c)**, IL-17A **(d)**, and RORγt **(e)** in the proximal colon of mice treated with different engineered bacteria. Data are presented as mean values ± SEM (n = 6 biologically independent samples for **(a-e)**). P values determined by one-way ANOVA with post hoc Tukey’s correction for multiple comparisons for all groups (**P* < 0.05, ***P* < 0.01, ****P* < 0.001 and *****P*<0.0001).


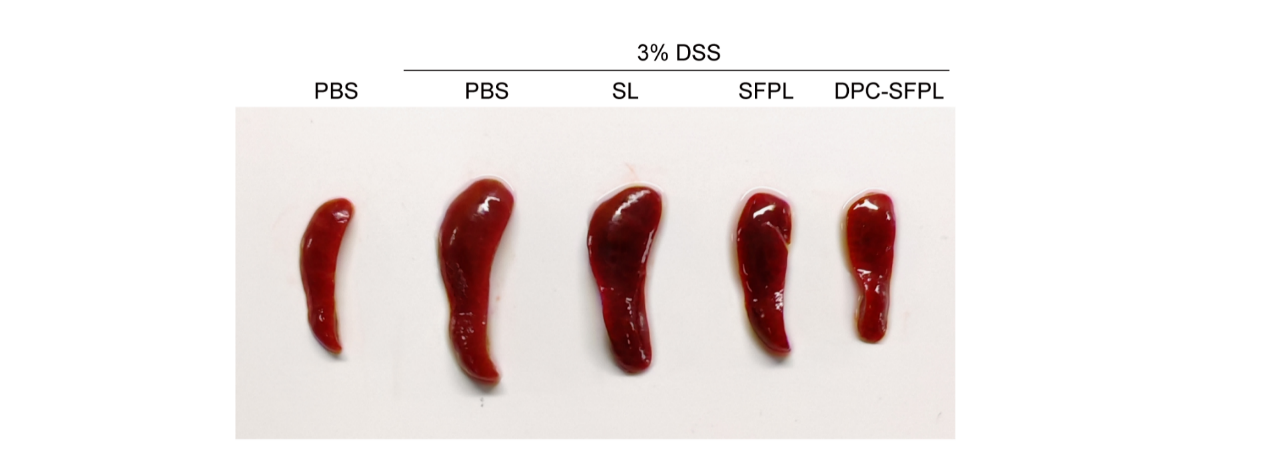
 **Fig. S22.** Images of spleen size in mice volumes with different engineered bacteria treatments


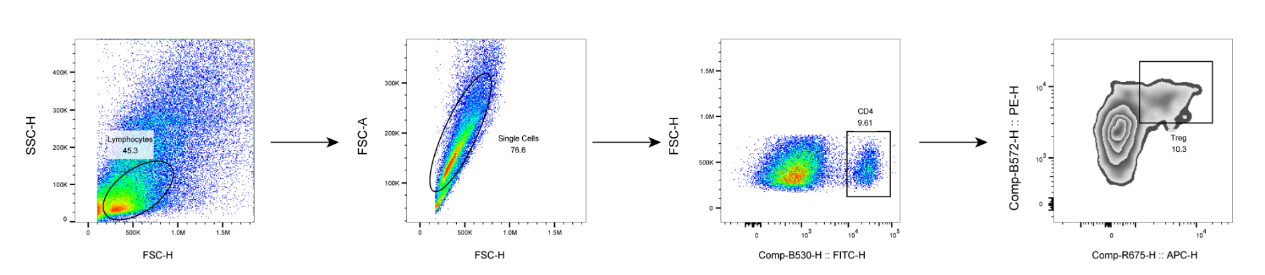


**Fig. S23.** Representative gating strategy used for flow cytometry analysis of Tregs in colonic lamina propria tissues.


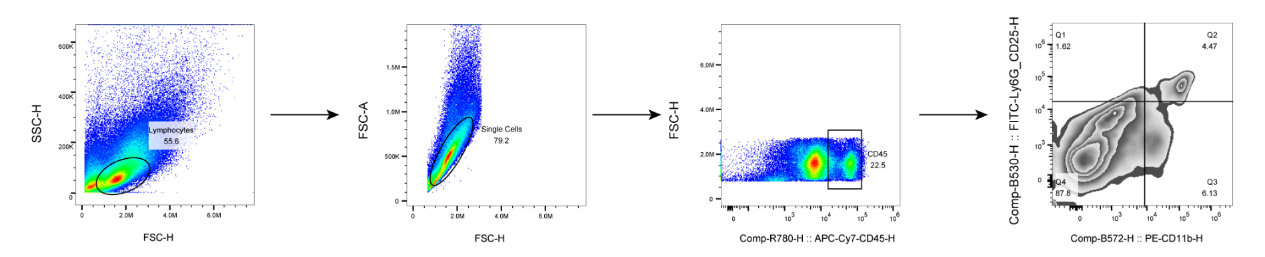


**Fig. S24.** Representative gating strategy used for flow cytometry analysis of Ly6G^+^CD45^+^CD11b^+^ cells in colonic lamina propria tiss*ues* of mice with different treatments.


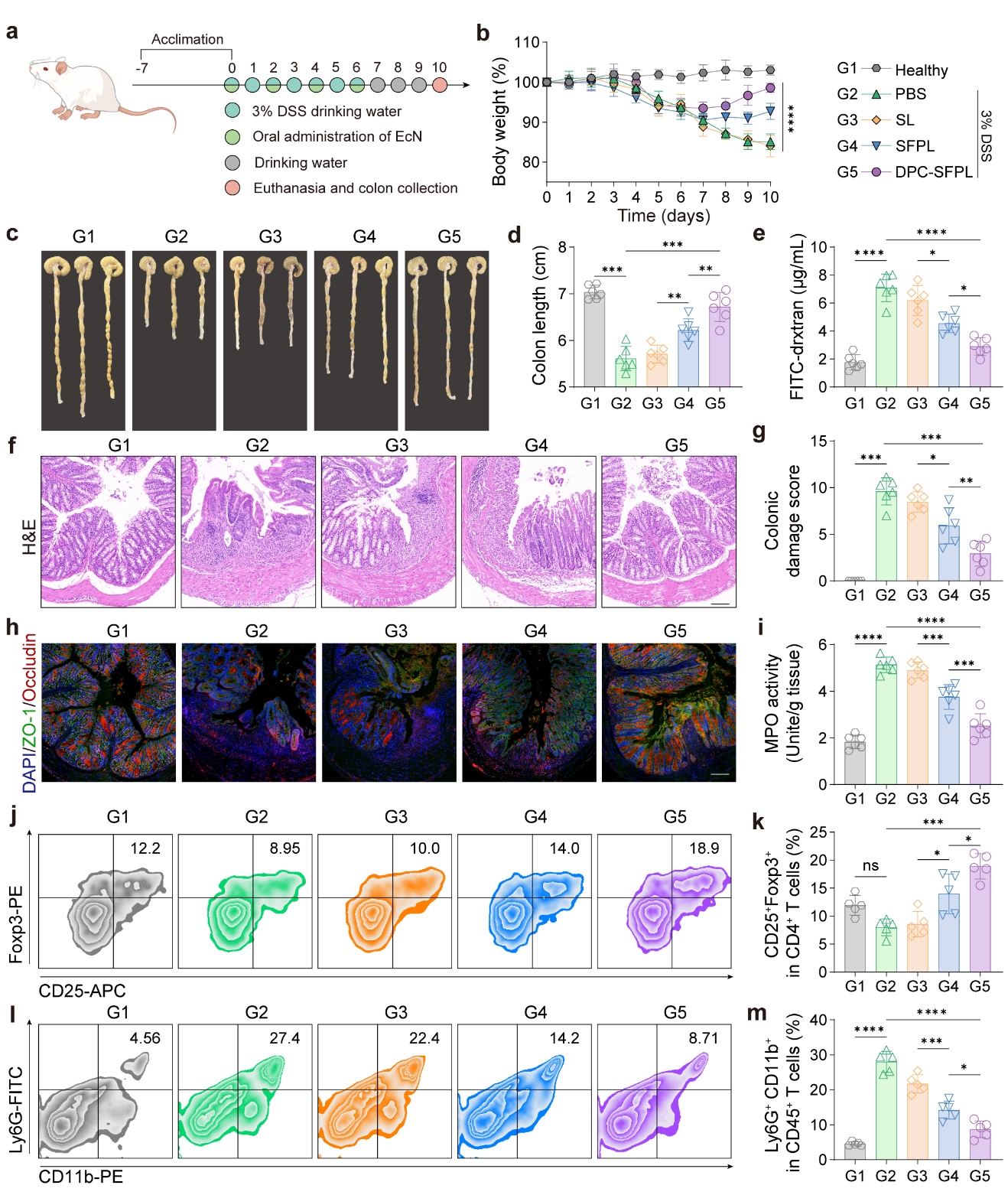
 **Fig. S25. a,** Schematic illustrating the experimental protocol for treating DSS-induced IBD mice. **b,** The weight of the mice was measured after receiving various treatments. **c-d,** Images **(c)** and lengths **(d)** of colons obtained from mice after different treatments on day 10. **e,** The assessment of intestinal integrity in mice following various treatments using FITC-dextran. **f,** H&E staining images of colon tissue samples obtained from mice with different treatments on day 10. Scale bar, 100 μm. **g,** The colonic damage scores of mice following various treatments. **h,** Image of immunofluorescence staining of the colon in a DSS-induced IBD model. ZO-1 (green), Occludin (red), and cell nuclei stained with DAPI, Scale bar, 100 μm. **i,** The MPO activity in the colons of mice after different treatments. MPO, myeloperoxidase. **j-m,** Flow cytometry analysis of Treg cells **(j)** (CD25^+^Foxp3^+^) and neutrophils **(l)** (CD11b^+^Ly6G^+^) in the colonic lamina propria of mice treated with different engineered bacteria, and statistical data on Tregs **(k)** and neutrophils **(m)**. Data are presented as mean values ± SEM (n = 5 biologically independent samples for **(k, m)**, n = 6 biologically independent samples for **(d-e, g, i)**). P values determined by one-way ANOVA with post hoc Tukey’s correction for multiple comparisons for all groups (**P* < 0.05, ***P* < 0.01, ****P* < 0.001 and *****P*<0.0001).


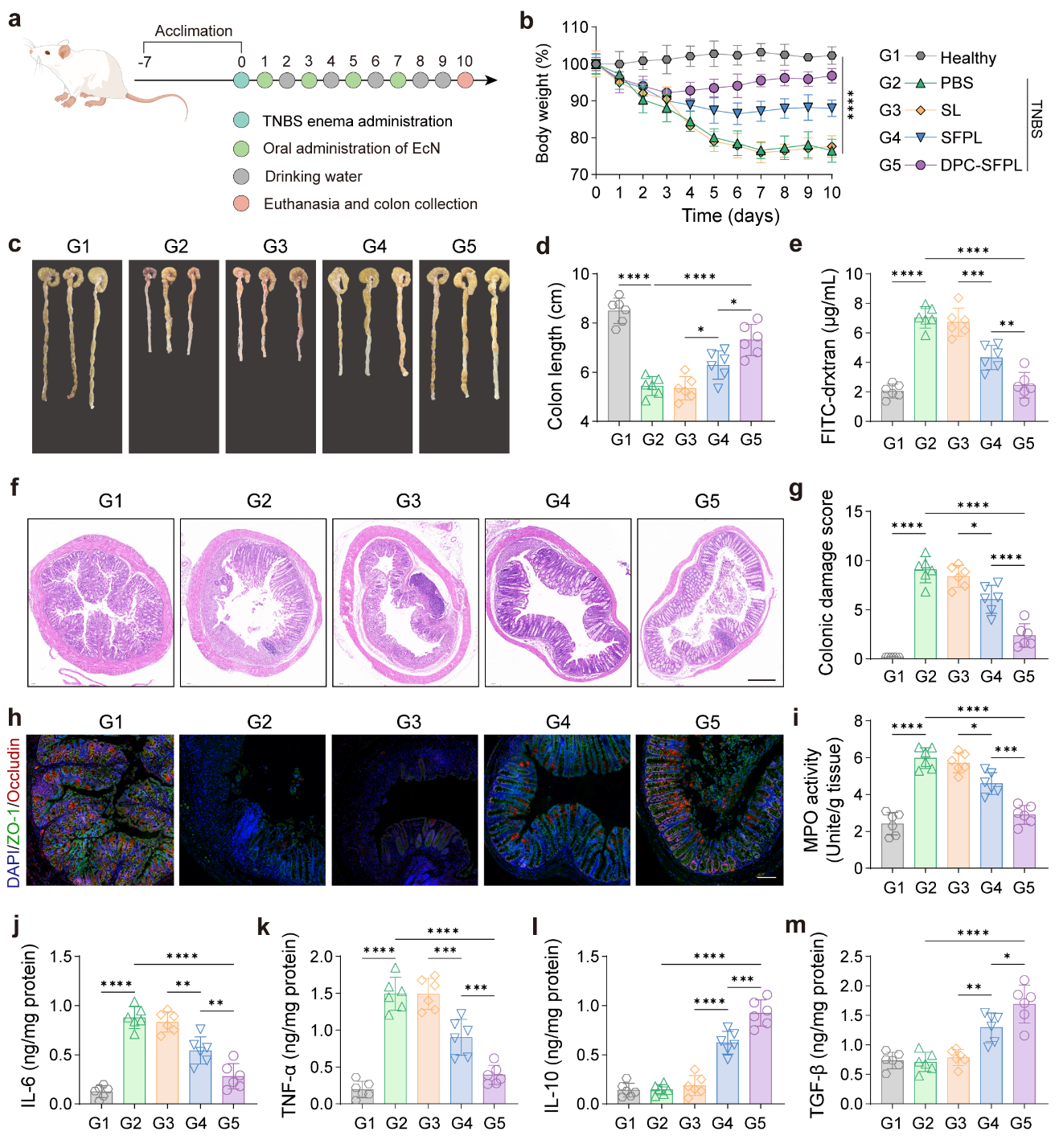


**Fig. S26. a,** Schematic illustrating the experimental protocol for treating TNBS-induced IBD mice. **b,** The weight of the mice was measured after receiving various treatments. **c-d,** Images **(c)** and lengths **(d)** of colons obtained from mice after different treatments on day 10. **e,** The assessment of intestinal integrity in mice following various treatments using FITC-dextran. **f,** H&E staining images of colon tissue samples obtained from mice with different treatments on day 10. Scale bar, 100 μm. **g,** The colonic damage scores of mice following various treatments. **h,** Image of immunofluorescence staining of the colon in a DSS-induced IBD model. ZO-1 (green), Occludin (red), and cell nuclei stained with DAPI, Scale bar, 100 μm. **i,** The MPO activity in the colons of mice after different treatments. MPO, myeloperoxidase. **j-m**, The levels of IL-6 **(j)**, TNF-α **(k)**, IL-10 **(l)**, and TGF-β **(m)** in the colon tissues measured by ELISA on day 10. Data are presented as mean values ± SEM (n = 5 biologically independent samples for (k, m), n = 6 biologically independent samples for (d-e, g, i, j-m)). P values determined by one-way ANOVA with post hoc Tukey’s correction for multiple comparisons for all groups (**P* < 0.05, ***P* < 0.01, ****P* < 0.001 and *****P*<0.0001).


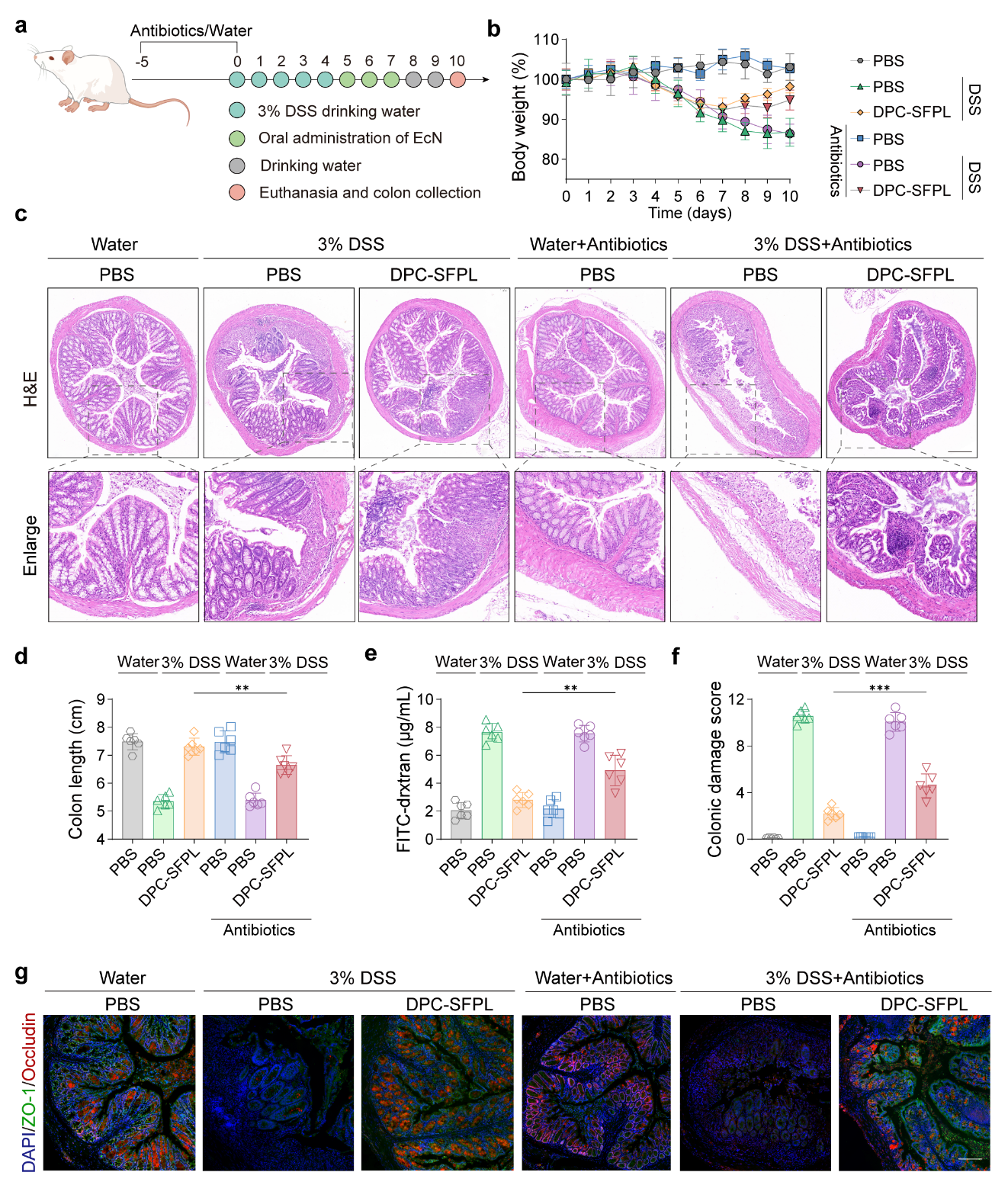


**Fig. S27. a,** Mice were successively exposed to 3 % DSS water, pre-treated with antibiotics, and orally intervened with DPC-SFPL, finally, colon samples were collected. **b,** the weight loss in the DPC-SFPL group was the smallest, significantly better than the DSS group and the combined antibiotic treatment group. **c,** H&E staining images of the colon after treatment with engineered bacteria in a DSS-induced IBD model in mice treated with antibiotics, Scale bar, 100 μm. **d-f,** colon shortening, decreased intestinal permeability, and significantly improved tissue damage scores. **g,** Image of immunofluorescence staining of the colon after treatment with DPC-SFPL engineered bacteria in a DSS-induced IBD model in mice treated with antibiotics. ZO-1 (green), Occludin (red), and cell nuclei stained with DAPI, Scale bar, 100 μm. DPC-SFPL (Dual plasmid compensation-SLC-Functional protein-L100). Data are presented as mean values ± SEM (n = 6 biologically independent samples for (b, d-f)). Statistical significance was calculated compared with the PBS group (d-f). P values determined by Student’s two-sided t-test. (**P* < 0.05, ***P* < 0.01, ****P* < 0.001 and *****P*<0.0001).


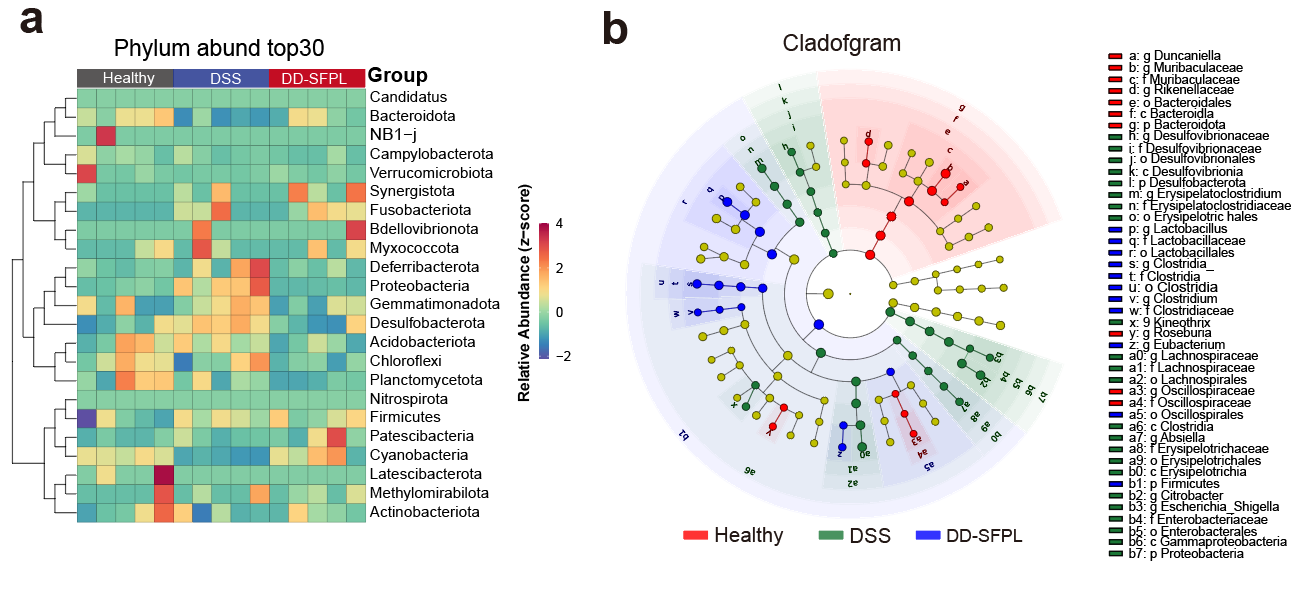


**Fig. S28. a-b** 16S rDNA sequencing analysis of gut microbiota. Heatmap showing relative abundance (z-score) of the top 30 microbial phyla **(a)** in Healthy, DSS, and DD-SFPL groups. Cladogram depicting taxonomic differences among the three groups **(b)**.

**Table S1. Plasmid construction primers**

| **Name** | **Sequence** |
| --- | --- |
| pKD3/ pKD4 Forward | tgtgtaggctggagctgcttcg |
| pKD3/ pKD4 Reverse | gccatggtccatatgaatatcctcct |
| pKD3-asd-Forward | CGGTAtGGTCGGCTCCGTTCTCATGCAACGCATGGTTGAAGAGCGCGAtgtgtaggctggagctgcttcg |
| pKD3-asd-Reverse | CGACGCAGCGGCTCCGCGGCCCCCCACAGCAGCTGGTCGCCCACGGTAgccatggtccatatgaatatcctcct |
| pKD4-thyA-Forward | GAACTGATGCAAAAAGTGCTCGACGAAGGCACACAGAAAAACGACCGTAtgtgtaggctggagctgcttcg |
| pKD4-thyA-Reverse | CGGCGCTTTAATGCCCGGATGCGGATCGTAGCCTTCAATCTCAAAGTCTTgccatggtccatatgaatatcctcct |
| 174E-Fragment-Forward | gaggagaaaggtaccATGGTACGCTGGACTTTGTGG |
| 174E-Fragment-Reverse | cagctaattaagcttTCACTCCTTCCGCACGTAATTTT |
| pTD103-Vector-Forward | aagcttaattagctgatctagacgcgt |
| pTD103-Vector-Reverse | ggtacctttctcctctttaa |
| pTD103-Vector-Forward-2 | taaggttgggaagccctgcaaagtaaactggatgg |
| pTD103-Vector-Reverse-2 | gatccttggcggcaagaaagccatccagtttactt |
| Double gene express-A-F | gccgcactcgagcacc |
| Double gene express-A-R | aagcttggcgtaactccttcttaa |
| Double gene express-B-F | ggatcctgataattaatttttccaggcatcaa |
| Double gene express-B-R | atcccgacccatttgctgtc |
| mcherry-Forward | gcaaatgggtcgggatatggtgagcaagggcgagga |
| mcherry-Reverse | ggtgctcgagtgcggccttgtacagctcgtccatgcc |
| GFP-Forward | agttacgccaagcttatggtgagcaaaggtgaagaactg |
| GFP-Reverse | taattatcaggatccttatttgtagagctcatccatgccgt |
| Amuc_1100-Forward | gacagcaaatgggtcgggatAGCAATTGGATTACAGACAACAAGCC |
| Amuc_1100-Reverse | ggtgctcgagtgcggcATCTTCAGACGGTTCCTGTGC |
| IL-2-1-Forward | GAGTTACGCCAAGCTTATGTACAGCATGCAGCTCGC |
| IL-2-1-Reverse | GCTGATGAAATTCTCAGCATCTTCCA |
| IL-2-2-Forward | GCTGAGAATTTCATCAGCCGGATCAGAGACACTGTTGTAAAACTAAAG |
| IL-2-2-Reverse | TTAATTATCAGGATCCTTACTTATCGTCGTCATCCTTGTAATCGCTGCCTTGAGGGCTTGTTGAGATGATG |

**Table S2. Disease activity index (DAI) parameters and associated scoring.**

| **Score** | **Weight loss (%)** | **Stool consistency** | **Blood in stool** |
| --- | --- | --- | --- |
| 0 | None | Normal | Normal |
| 1 | 1-5 | Slight loose stool | Small presence of blood |
| 2 | 5-10 | Loose stool | Significant presence of blood |
| 3 | 10-15 | Diarrhea | Gross blood |
| 4 | ＞15 |  |  |

**Table S3. Histological grading scheme for DSS colitis.**

| **Colonic epithelial damage** | | **Inflammatory cell infiltration** | |
| --- | --- | --- | --- |
| Score | Description | Mucosa | |
| 0 | Normal | Score | Description |
| 1 | Hyperproliferation,irregular crypts, and goblet cell loss; | 0 | Normal |
| 2 | Mild to moderate crypt loss (10 -50%) | 1 | Mild |
| 3 | Severe crypt loss (50-90%) | 2 | Modest |
| 4 | Complete crypt loss, surface epithelium intact | 3 | Severe |
| 5 | Small-to medium-sized ulcers (＜10 crypt widths) | Submucosa | |
| 6 | Large ulcers (≥10 crypt widths) | Score | Description |
|  |  | 0 | Normal |
|  |  | 1 | Mild to modest |
|  |  | 2 | Severe |
|  |  | Muscle/serosa | |
|  |  | Score | Description |
|  |  | 0 | Normal |
|  |  | 1 | Moderate to severe |

**Table S4. qPCR Primer**

| **Gene** | **Forward sequence** | **Reverse sequence** |
| --- | --- | --- |
| IL-6 | TAGTCCTTCCTACCCCAATTTCC | TTGGTCCTTAGCCACTCCTTC |
| Foxp3 | GGGGAAGCCATGGCAATAGT | GGCATAGGTGAAAGGGGGTC |
| IL-17A | GCTCTGCTTCTGGGGACTTT | GGGTCTGCACAGATGAGCTT |
| RORγt | ACAATGGTCTCCAGAGGGTG | ATTCGGCAATGTGGTCAAAC |
| Muc2 | AGGGCTCGGAACTCCAGAAA | CCAGGGAATCGGTAGACATC |
| Occludin | ACGTCCGACCCATGCTCTCT | AAGTCATCCGCAGGGGAGGT |
| ZO-1 | TGGTCTGTTTGCCCACTGTT | TCTGTACATGCTGGCCAAGG |

**Table S5.** Reagent or resource

| **Reagent or resource** | **Source** | **Identifier** |
| --- | --- | --- |
| **Antibodies** | | |
| APC/Cy7 anti-mouse CD45 | Biolegend | 103116 |
| PE anti-mouse CD11b | Biolegend | 101208 |
| FITC anti-mouse LY6G | Biolegend | 127605 |
| PERCP5.5 anti-mouse CD4 | Biolegend | 100434 |
| APC anti-mouse CD25 | Biolegend | 101909 |
| Anti-rabbit antibody 647 | Invitrogen | A31573 |
| Anti-mouse antibody 488 | Invitrogen | A11001 |
| Anti-FLAG tag-HRP | Beyotime | AF2855 |
| Anti-HIS tag-HRP | Beyotime | AF2879 |
| MUC2 Antibody | Affinity | DF8390 |
| ZO-1 Antibody | Affinity | AF5145 |
| Occludin | Affinity | DF7504 |
| **Chemicals and proteins** | | |
| DMEM | KETU Biotechnolog | RG-CE-5 |
| FBS | Gibco | A5256701 |
| Penicillin | Beyotime | ST486 |
| Streptomycin | Beyotime | ST487 |
| SDS-PAGE | EpiZyme | PG112 |
| LB basal medium | Solarbio | 20240522 |
| BSA | Biosharp | 9048-46-8 |
| Tween-20 | Biosharp | 9005-64-5 |
| Nonfat powdered milk | Beyotime | P0216 |
| BeyoECL Plus | Beyotime | P0018S |
| DSS | MeilunBio | 9011-18-1 |
| FITC-dextran | Beyotime | ST2940 |
| Protein Extraction Kit | Beyotime | P0013Q |
| diaminopimelic acid | Sigma | D1377 |
| thymidine | Sigma | T1895 |
| kanamycin | Beyotime | ST101 |
| chloramphenicol | Sigma | C0378 |
| L-(+)-Arabinose | Sigma | A3256 |
| Premier DNA Polymerase | Takara | RR371S |
| Plasmid Extraction Kit | Tiangen | A1226B |
| DNA Recovery Kit | Tiangen | Y1817 |
| RT SuperMix for qPCR | Vazyme | R222-01 |
| Real Time (TB Green Plus) | Takara | R075A |
| **Elisa assays** | | |
| Mouse TNF-α ELISA | Boster | EK0527 |
| Mouse IL-10 ELISA | Boster | EK0417 |
| Mouse IL-6 ELISA | Boster | EK0411 |
| Mouse TGF-β ELISA | Boster | EK0515 |
| MPO | Nanjing Jiancheng | A044-1-1 |
| **Mice** | | |
| BALB/c | GemPharmatech Co., Ltd | \ |
